# Faculty-supervised AI-supported BOPPPS-CBL and performance on a simulated neurological-emergency assessment among junior-college medical interns: a batch-stratified randomized educational study

**DOI:** 10.3389/fmed.2026.1945397

**Published:** 2026-10-14

**Authors:** Junhuai Zhang, Zhanqin Xiao, Xiang Gu, Qi Lang, Chanxi Chen, Yaxi Chen

**Affiliations:** 1 Holistic Center for Neurological Diseases, The People’s Hospital of Chongqing Yubei District, Chongqing, China; 2 School of Clinical Medicine, Chongqing Medical and Pharmaceutical College, Chongqing, China

**Keywords:** artificial intelligence, BOPPPS, clinical reasoning, medical education, neurology, simulation

## Abstract

**Background:**

County hospitals and township health centers in China provide frontline care for time-sensitive neurological emergencies, and three-year junior-college clinical medicine programs are an important pathway into this workforce. We evaluated a faculty-supervised artificial intelligence (AI)-supported BOPPPS (Bridge-in, Objectives, Pre-assessment, Participatory Learning, Post-assessment, and Summary) and case-based learning (CBL) instructional package among junior-college medical interns.

**Methods:**

In this prospective, batch-stratified randomized educational study, 118 interns from 12 monthly rotation batches were assigned to AI-supported BOPPPS-CBL (*n* = 58) or conventional BOPPPS-CBL (*n* = 60) during a one-month neurology rotation. The intervention used standardized DeepSeek prompts for Socratic questioning, error-focused feedback, and structured reflection under faculty supervision. The primary endpoint was the total score on a locally developed four-station simulated neurological-emergency assessment (0–100). Secondary outcomes included a written examination, while learner-reported questionnaire items were exploratory. Regression models adjusted for rotation batch, baseline academic performance, and digital literacy. The study was not prospectively registered; no public trial registration identifier or registry URL exists, and no publicly time-stamped protocol or statistical analysis plan was available.

**Results:**

All 118 participants completed the primary assessment. Mean total scores were 82.07 in the AI-supported group and 76.43 in the control group, with an adjusted between-group difference of 5.67 points (95% CI, 2.86–8.49; *P* < 0.001; adjusted Cohen's *d* = 0.74). In secondary station-specific analyses, only lesion localization met the multiplicity-adjusted threshold (adjusted mean difference, 2.58 points; 95% CI, 1.25–3.91; *P* < 0.001; adjusted *d* = 0.71). A Station 2-specific ICC was not estimated. *Post hoc* sensitivity analyses excluding the station containing reused case contexts yielded standardized estimates of similar magnitude but could not exclude cueing or carry-over. Written-examination estimates were smaller and imprecise. Exploratory learner-reported findings indicated greater perceived preparation-time burden in the AI-supported group.

**Conclusion:**

Assignment to the faculty-supervised AI-supported BOPPPS-CBL package was associated with higher scores on a locally developed simulated neurological-emergency assessment than conventional BOPPPS-CBL. These findings do not establish transfer to workplace clinical performance or patient outcomes. Multicenter studies using independent assessment cases, longer-term follow-up, and workplace-based outcomes are warranted.

## 1 Introduction

Disorders affecting the nervous system are major contributors to death and disability worldwide (1). Stroke illustrates the population-level stakes: in mainland China, the estimated 2020 incidence and mortality rates among adults aged 40 years or older were 505.2 and 343.4 per 100,000 person-years, respectively, with higher incidence and mortality in rural than in urban areas (2). In acute ischemic stroke, treatment delay is associated with rapid loss of nervous tissue, underscoring the need for emergent evaluation and therapy (3).

Lower-level health facilities carry a substantial share of this frontline responsibility. In 2023, county-level hospitals and township health centers in China delivered approximately 1.44 billion and 1.31 billion outpatient visits, respectively. Township health centers employed about 1.61 million health workers, including 572,000 licensed or assistant physicians (4). Three-year junior-college medical education is intended to prepare graduates for primary-care practice (5). Rural grassroots doctors nevertheless report persistent difficulties in integrating theory with clinical practice (6). Graduation-internship training is therefore relevant to preparing future clinicians to recognize, stabilize, and refer neurological emergencies in district, county, and township settings.

The immediate training challenge is not simply knowledge acquisition. Interns may recall disease names, diagnostic criteria, and the steps of a neurological examination yet struggle to apply this knowledge to clinical cases (7, 8). Relevant clinical-reasoning challenges include inferring lesion location from patterns of symptoms and signs, recognizing red flags, prioritizing emergency actions, and linking examination findings to referral decisions. These difficulties reflect deficits in higher-order clinical reasoning that are especially consequential when specialist support is limited and transfer decisions are time-sensitive.

The BOPPPS model (Bridge-in, Objectives, Pre-assessment, Participatory Learning, Post-assessment, and Summary) provides a structured six-phase framework for organizing active teaching (9), while case-based learning (CBL) engages learners in authentic, context-based clinical reasoning tasks. Their combination has been associated with improved engagement and learning outcomes in clinical education (10). However, in resource-constrained district hospitals, limited faculty time can make it difficult to provide each learner with timely, individualized, error-specific feedback. Whole-group discussion may therefore leave important misconceptions or reasoning gaps unaddressed.

Large language models may support scalable formative scaffolding by generating individualized prompts, feedback, and opportunities for structured reflection (11–13). However, much of the existing medical education literature has focused on feasibility, learner perceptions, satisfaction, or knowledge-based outcomes, while evidence based on directly observed clinical-reasoning performance remains comparatively limited (14, 15). Evidence from district-hospital and junior-college settings is also sparse. In the present study, AI was restricted to formative use: it did not reveal keyed answers before learners committed an initial response, contribute to grading, replace faculty judgment, or process identifiable patient information.

Self-regulated learning (16) and instructional scaffolding (17) provided the theoretical basis for the intervention. By making intermediate reasoning steps explicit and prompting learners to monitor and revise their thinking, the scaffold was intended to support these processes during localization, red-flag recognition, and triage. We therefore evaluated a faculty-supervised AI-supported BOPPPS-CBL instructional package, compared with conventional BOPPPS-CBL, among junior-college medical interns during a district-hospital neurology rotation. The primary endpoint was the total score on a locally developed four-station simulated neurological-emergency assessment. Secondary and exploratory outcomes included written-examination performance, learner-reported feedback-responsive behaviors, satisfaction, perceived implementation burden, and intervention-specific AI experience. We focused on between-group effect estimates and their uncertainty and interpreted the findings as educational performance outcomes rather than evidence of transfer to independent clinical practice.

The present study was distinct from our earlier randomized educational study conducted at the same institution, which evaluated an evidence-based-medicine-integrated BOPPPS model among three-year junior-college medical clerks from July 2024 to May 2025 (18). The present study enrolled a completely non-overlapping cohort of medical interns from July 2025 to June 2026 and evaluated faculty-supervised AI-supported scaffolding. Its four-station assessment was newly developed and was not adapted from or reused from the earlier modified OSCE; cases, teaching faculty, standardized patients, and assessors also did not overlap. Because the instruments were developed independently and were not formally equated, their absolute scores should not be compared directly.

## 2 Methods

### 2.1 Study design and reporting

This prospective, two-arm, batch-stratified, parallel-group randomized educational study compared AI-supported BOPPPS-CBL with conventional BOPPPS-CBL. Each consecutive monthly rotation batch formed a randomization stratum, and participants were individually randomized in near-equal proportions within each batch. The study was not prospectively registered, and no public trial registration identifier or registry URL exists. Internal study-planning drafts were repeatedly revised in place; no unaltered, independently date-verifiable version of a study-specific protocol or statistical analysis plan from before enrollment or outcome examination was retained. The current drafts therefore cannot establish prespecification. Consequently, the chronology of outcome prioritization and analytical decisions cannot be independently verified. Analyses developed after examination of the study data are identified as *post hoc*. Supplementary Table S1 summarizes the reported outcome and analysis hierarchy but does not establish prespecification. Reporting was guided by CONSORT 2025 and TIDieR, while the reporting and interpretation of the AI-supported educational intervention were informed by methodological guidance for AI research in medical education (19–21).

### 2.2 Setting and participants

The study was conducted in the Department of Neurology at The People's Hospital of Chongqing Yubei District, Chongqing, China, from July 2025 to June 2026. Interns rotated through the department in 12 consecutive monthly batches during one academic year. Ten batches included 10 interns and two included 9 interns, yielding an annual cohort of 118. Eligible participants were three-year junior college clinical medicine students from Chongqing Medical and Pharmaceutical College who were completing their graduation internship in neurology, had completed the required foundational courses, provided written informed consent, and could attend the scheduled rotation. Pre-randomization exclusion criteria were previous systematic AI-assisted neurology training or withdrawal of consent before allocation. After randomization, absence, incomplete exposure, or missing outcome data did not constitute grounds for exclusion from the intention-to-treat population. Such events were to be documented, and participants were retained in the analysis when outcome data were available.

### 2.3 Randomization, allocation concealment, and baseline comparability

An independent statistician who was not involved in recruitment, teaching, or outcome assessment generated a computer-based allocation sequence stratified by monthly batch. Each 10-person batch was allocated equally between groups. In each 9-person batch, the concealed sequence assigned four interns to the AI-supported group and five to the control group, producing final group sizes of 58 and 60, respectively. A research administrator with no teaching or scoring role implemented allocation after all participants in a batch had completed enrollment and baseline assessment. Recruiters, teachers, and assessors had no access to future assignments. Baseline measures included age, gender, clinically relevant academic performance, digital literacy, and generative-AI use. Baseline clinically relevant academic performance was calculated from the participants’ Diagnostics and Neurology course scores obtained in the semester immediately preceding the clinical internship. Both courses were graded on a 0–100 scale, and the composite score was calculated as their credit-weighted mean. Digital literacy was assessed using a study-developed five-item measure rated from 1 to 5. A mean score was calculated when at least four items were completed. Generative-AI use for medical learning or clinical case analysis during the previous three months was categorized as never, occasional (<1 time/week), or frequent (≥1 time/week). Baseline characteristics were summarized using standardized effect sizes without significance testing. Adjusted outcome models included rotation batch, baseline academic performance, and digital literacy.

### 2.4 Sample size

The study sample was determined by the available rotation cohort during the study period. All 118 eligible interns were invited to participate. No *a priori* sample-size calculation was performed, and no minimal important difference for the OSCE total score was prospectively specified. Because randomization was conducted at the individual level within rotation batches rather than at the cluster level, no cluster design-effect adjustment was required. Effect estimates are therefore presented with 95% confidence intervals to emphasize their magnitude and precision.

### 2.5 Intervention: AI-supported BOPPPS-CBL

The intervention was delivered during the one-month neurology rotation as six structured modules. Two 50–60-minute scheduled sessions were held each week during the first three weeks; the fourth week was reserved for integration, review, and end-of-rotation assessment. The modules addressed: (1) acute ischemic stroke recognition and triage; (2) hemorrhagic stroke and acute severe headache; (3) acute dizziness and differentiation of central from peripheral vertigo; (4) seizure, status epilepticus, and altered consciousness; (5) acute limb weakness, numbness, and lesion localization across the neuraxis and neuromuscular system; and (6) integration of the neurological examination with referral decision-making. The planned six-session schedule and attendance are summarized in Supplementary Table S2. Both groups were scheduled for six 50–60-minute sessions, corresponding to 300–360 min of planned contact time per participant.

AI was embedded as a formative scaffold across the BOPPPS sequence, as summarized in Figure 2 and Supplementary Table S3. The standardized Chinese prompt templates and English reporting versions are provided in Supplementary Appendix S1. Learners were required to commit an independent initial response before receiving AI feedback. During bridge-in and pre-assessment, prompts directed attention to onset, time windows, red flags, supporting and conflicting findings, and alternative interpretations. During participatory learning, the AI used one-question-at-a-time Socratic prompts to support localization and triage reasoning. During post-assessment, it provided individualized, non-graded feedback structured around strengths, omissions, and priorities for improvement. During summary, learners first generated their own localization maps, red-flag checklists, triage pathways, initial-management checklists, and reflections before using AI to refine their organization. Formal marking and authoritative clinical explanations remained the responsibility of faculty.

DeepSeek was accessed through its public web or mobile application interface. A standardized prompt sequence dated January 2025 was used throughout the intervention. Because the interface-displayed model version and optional platform settings could change over time, the intervention should be interpreted as a prompt- and faculty-supported instructional package rather than as an evaluation of a fixed model build. Faculty monitored learner interactions and were instructed to intervene when outputs appeared premature, inaccurate, unsafe, inconsistent with the local pathway, or beyond the intended formative role of the AI. Clinical decisions involving specific treatment regimens, procedural timing, contraindications, or local pathway requirements remained under faculty and guideline authority. The study did not retain a standardized session-level log of model version or configuration, number of prompts, completion of each scaffold stage, premature disclosure of answers, inaccurate or unsafe outputs, or faculty correction events. These dimensions of intervention fidelity therefore cannot be quantified retrospectively. Scheduled teaching time was matched between groups. However, minutes spent in AI dialogue, reflection, additional learner preparation outside scheduled sessions, and activity-specific faculty interaction were not recorded. Thus, the delivered activity-specific dose cannot be quantified or compared.

### 2.6 Comparator: conventional BOPPPS-CBL

The control group received the same six modules, learning objectives, scheduled teaching frequency and duration, and faculty-matched case content and difficulty through conventional BOPPPS-CBL. Learners completed the same types of initial case responses, case discussions, formative exercises, and learning summaries, but feedback was provided by faculty and peers without access to the standardized AI prompt templates. The control group therefore represented an active educational comparator rather than usual teaching without structured instruction. The same amount of faculty contact time was scheduled in both groups, but activity-specific faculty minutes and unscheduled learner preparation time were not measured. Accordingly, the randomized contrast estimates the effect of assignment to the AI-supported instructional package under the study conditions and does not isolate the effect of AI-generated feedback itself.

### 2.7 Implementation procedures and contamination control

All participating faculty completed a calibration workshop and followed a shared teaching manual. The two groups were taught in separate rooms on nonoverlapping schedules. Intervention prompt templates and answer materials were not distributed to control participants, and participants were asked not to share study materials before completion of the final assessments. Assessment cases, wording, images, videos, and standardized-patient scripts were not used in teaching, formative quizzes, AI prompt demonstrations, or practice sessions. Attendance was recorded for every scheduled session. In the intervention group, use of the AI-supported approach during the rotation was recorded as an implementation-coverage measure. In the control group, potential study-related generative-AI use during the six modules was assessed by participant self-report. The exact wording of the monitoring question, its precise administration time, and its response options were not retained. The self-report measure did not systematically distinguish study-related use from unrelated use of other AI tools or cross-group sharing. No independent digital-use monitoring, device audit, or platform log was available. Consequently, the finding can be described only as no reported study-related use, not as verified absence of contamination. No formal intervention-fidelity score, complete batch-level implementation dataset, or independent implementation audit was available.

### 2.8 Blinding

Learners and faculty could not be blinded to group assignment because of the nature of the intervention. Participants assigned to the intervention group were aware that they were receiving a novel AI-supported instructional package, and faculty in that arm additionally monitored AI interactions and corrected inaccurate or unsafe outputs. Although scheduled teaching duration was matched between groups, these differences could have influenced learner expectations, effort, preparation time, faculty attention, or other behaviors independently of the content of AI-generated feedback. Learner expectations were not measured. The allocation sequence remained concealed until each participant had been assigned. OSCE assessors were blinded to group assignment, and written response sheets and station records were identified only by study code. Whether assessors or standardized patients could infer group assignment from learner behavior was not formally assessed. The primary treatment estimate is therefore interpreted as the effect of randomized assignment to the AI-supported instructional package rather than as an isolated causal effect of AI-generated feedback.

### 2.9 Ethics and consent

The study was conducted in accordance with the 2024 revision of the Declaration of Helsinki. The underlying teaching-reform project was reviewed by the Ethics Committee of The People's Hospital of Chongqing Yubei District, Chongqing, China. The project documentation described AI-assisted teaching as one component at a general level, without specifying a particular platform or model. Following an expedited review procedure, the Ethics Committee determined that the project met the criteria for exemption from formal ethics review because it involved the application of teaching methods and learning-outcome assessment in a normal educational setting, used only simulated or fully de-identified cases, involved no interventional procedures with patients, and had no commercial interests. The Committee issued an exemption determination documented as Ethics Review Opinion No. 2023C03 on 22 November 2023. Because this was an exemption determination rather than full ethics approval, no separate ethics approval number was issued. The present study represented a more detailed implementation and evaluation of the AI-assisted teaching component within this teaching-reform framework. Participation was voluntary, and written informed consent was obtained before enrollment, including consent to randomized allocation and the collection and analysis of de-identified educational data. Participation, group allocation, and AI use did not affect official grading criteria or internship evaluation, and participants could withdraw without academic penalty. Consent was obtained by research personnel who were not responsible for participants’ academic grading. Control-group participants received access to the standardized AI-scaffolding materials after completion of all study outcome assessments. At the time of study implementation, the institution had general requirements concerning research ethics, confidentiality, and educational research but did not yet have a dedicated ethics-review pathway or standardized documentation requirements specifically for the use of public generative-AI platforms in medical education. Accordingly, the exemption determination should not be interpreted as a technical audit or institutional endorsement of the DeepSeek platform. The public DeepSeek interface was used only with simulated or fully de-identified educational cases. Learners were instructed not to enter names, identification numbers, contact information, medical-record numbers, identifiable images, or other directly identifiable patient information. Study assessment data, allocation information, and participant identifiers were stored separately on access-controlled institutional systems and were not entered into the AI interface. The study did not retain sufficient contemporaneous documentation to reconstruct the model version and account-level settings, third-party data-retention or secondary-use settings, model-training settings, or data-processing locations applicable to every session. These platform-level characteristics are therefore not inferred retrospectively. The principal safeguards were data minimization, separation of research data from AI interactions, restriction to simulated or fully de-identified cases, and faculty supervision of AI-supported learning.

### 2.10 Primary outcome

The primary outcome was the post-rotation total score on a locally developed four-station structured simulated neurological-emergency assessment (0 -100, with 25 points per station), comprising three written vignette stations and one standardized-patient station and referred to hereafter as the objective structured clinical examination (OSCE). The complete assessment blueprint and scoring rubrics are provided in Supplementary Appendix S2. Station 1 used three short vignettes to assess red-flag recognition. Station 2 used two vignettes to assess lesion localization. Station 3 used two vignettes to assess disposition, immediate action, and time-critical referral. Within each parallel form, Station 3 reused two anchor cases previously encountered in Station 1 to assess a different reasoning domain. Stations 2 and 4 did not share assessment cases with each other. Station 4 assessed a focused neurological examination on a standardized patient. Findings that could not be simulated reliably, including pathological plantar responses, direction-changing nystagmus, skew deviation, and head-impulse test results, were supplied only after the candidate demonstrated or requested the relevant maneuver, using standardized result cards or video clips. Each station comprised an 18-point analytic checklist and a 7-point anchored global rating. Two assessors blinded to allocation scored each station independently, and their scores were averaged. Two parallel assessment forms were alternated across consecutive monthly rotation batches. Form A was administered in batches 1, 3, 5, 7, 9, and 11, whereas Form B was administered in batches 2, 4, 6, 8, 10, and 12. Both randomized groups within each batch completed the same form.

### 2.11 Secondary outcomes

The secondary performance outcome was a 100-point end-of-rotation written examination described in Supplementary Appendix S3. It comprised 25 single-best-answer multiple-choice questions (MCQs; 0–50 points) and five key-feature-question (KFQ) cases (0–50 points). Questionnaire outcomes were treated as exploratory. All participants completed 14 post-rotation items: eight on self-regulated learning behaviors, three on satisfaction, and three on perceived learning burden. Participants assigned to the AI-supported instructional package additionally completed 12 items addressing perceived usefulness, perceived ease of use, trust in AI feedback, possible reliance on AI prompting, and future use intention. All questionnaire items used five ordered response categories ranging from 1 (strongly disagree) to 5 (strongly agree). The 12 intervention-only items were summarized descriptively and were not used for between-group comparisons. Complete item wording and scoring information are provided in Supplementary Appendix S4.

### 2.12 Measurement development, pilot testing, and reliability

The four-station structured simulated neurological-emergency assessment was newly developed specifically for this study and was not adapted from or reused from the modified OSCE in our earlier EBM-BOPPPS study (18). It underwent expert content review by five neurologists, who examined its relevance, clarity, clinical accuracy, and alignment with the intended constructs. In May 2025, 15 learners participated in feasibility pilot testing that examined administration, score distributions, floor and ceiling patterns, apparent differences between the two assessment forms, and scoring clarity; no change to the scoring rubric was required. These procedures constituted expert content review and feasibility testing rather than formal psychometric validation. Before the assessment, all assessors completed a calibration workshop using common written or video samples. Inter-rater reliability was quantified using a two-way random-effects, absolute-agreement intraclass correlation coefficient for the mean of two assessors [ICC(2,2)]. Reliability was estimated for the total assessment score and for station-level ratings pooled across all four stations; separate station-specific ICCs were not estimated. As defined in the scoring protocol, a difference greater than 4 points between the two assessors on any station would have triggered independent scoring by a third blinded assessor. Internal consistency across the four intentionally distinct stations was summarized descriptively using Cronbach's alpha. The MCQ and KFQ components of the written examination were pilot-tested for item difficulty, discrimination, score distribution, scoring clarity, and, where applicable, inter-rater agreement.

The five-item baseline digital-literacy measure was a study-specific self-report instrument administered in Chinese. It covered device proficiency, medical-information retrieval, evaluation of online medical content, digital communication, and openness to new digital tools. Items were rated from 1 to 5, and the mean of the five items was used as the digital-literacy score, with higher scores indicating greater self-reported digital literacy. Item-level responses were aggregated at data entry and were not retained in the analytic dataset; consequently, item-level distributions and internal consistency for this measure could not be reconstructed *post hoc*.

The post-rotation questionnaires were also study-specific and were administered in Chinese. The items underwent cognitive interviewing and feasibility pilot testing before use; these procedures were intended to improve wording clarity and administration and were not regarded as formal construct validation. The questionnaires were not treated as implementations of previously validated multi-item scales. Supplementary Appendix S4 presents the English reporting version. The available study records do not document a formal independent forward-translation, back-translation, or linguistic-validation procedure for this English rendering, which was not used to collect participant responses. Given the limited validity evidence and the low or negative internal-consistency estimates observed in the trial sample, questionnaire findings were treated as exploratory at the item level, and no domain-level composite scores were interpreted.

### 2.13 Data collection

Baseline demographic variables, Diagnostics and Neurology course scores obtained during the semester immediately preceding the clinical internship, digital literacy, and recent generative-AI use were collected before allocation was disclosed. Outcome assessment was conducted after the final teaching session. Within the four-station simulated neurological-emergency assessment, the three written-vignette stations and the live standardized-patient station were completed on the same day in a single assessment sitting and in the same fixed sequence for all participants. Each station lasted 10 min, with 2 min for transfer and instructions. Apart from this fixed station sequence and common timing, the available study records do not document an additional counterbalancing procedure or a formal assessment of candidate-order, assessor-order, or fatigue effects. The 100-point written examination and the post-rotation questionnaire were administered after the final teaching session and before control participants received access to the AI materials. All participants completed the self-regulated-learning, satisfaction, and perceived-burden items, and participants assigned to the AI-supported instructional package additionally completed the 12 AI-experience items. Attendance was recorded for each of the six scheduled teaching sessions. Intervention-group use of the AI-supported approach and control-group use of generative AI for the study modules were assessed by self-report during the rotation. No digital usage logs or device-level audits were available; therefore, unreported use or cross-group sharing could not be excluded. Assessment materials were identified by study code and contained no information on randomized assignment. Before analysis, the dataset underwent predefined range and internal-consistency checks.

### 2.14 Statistical analysis

The primary endpoint was the post-rotation four-station OSCE total score. The primary estimand was the intention-to-treat effect of randomized assignment to the AI-supported instructional package, compared with conventional BOPPPS-CBL, on this score. The primary analysis used linear regression with treatment group and rotation-batch fixed effects, adjusted for baseline academic performance and digital literacy. Results are reported as adjusted mean differences, 95% confidence intervals, two-sided *P* values, and adjusted Cohen's d. Adjusted Cohen's d was calculated by dividing the adjusted mean difference by the residual standard deviation from the corresponding regression model. Linear-regression assumptions were assessed by inspection of residual distributions and residual-versus-fitted plots. The written-examination total score and its MCQ and KFQ components were analyzed using analogous linear-regression models and were treated as secondary educational outcomes. No family-wise multiplicity adjustment was applied to these three written-examination outcomes; interpretation therefore emphasized effect estimates and 95% confidence intervals rather than confirmatory significance claims.

Each of the 14 common post-rotation questionnaire items was analyzed separately using proportional-odds logistic regression with treatment group, rotation batch, baseline academic performance, and digital literacy. Adjusted odds ratios greater than 1 indicate higher odds of selecting a higher response category in the AI-supported group. The proportional-odds assumption was assessed for each model using the test of parallel lines, and model convergence was reviewed. Because the study-developed questionnaire domains showed inadequate internal consistency, composite scores were not interpreted. The eight self-regulated-learning items were treated as one exploratory outcome family, with a Bonferroni-adjusted threshold of *P* < 0.00625. The three satisfaction items and three perceived-burden items were treated as exploratory learner-reported outcomes without family-wise multiplicity-adjusted confirmatory claims.

Because several original Likert response categories were sparse or empty, a sensitivity analysis collapsed the five response categories into three ordered categories: disagree (scores 1–2), neutral (score 3), and agree (scores 4–5). The proportional-odds models were then repeated using the same covariates. For SAT02, the collapsed-category model showed evidence against the proportional-odds assumption; a supplementary binary logistic-regression sensitivity analysis therefore compared agree/strongly agree (scores 4–5) with all other responses (scores 1–3), using the same adjustment variables. These questionnaire sensitivity analyses were exploratory. The 12 intervention-only AI-experience items were summarized descriptively. Exploratory item groupings for perceived usefulness, ease of use, trust in AI feedback, possible reliance on AI prompting, and future use intention were summarized using the mean, standard deviation, median, interquartile range, and Cronbach's alpha. These groupings were not interpreted as validated constructs and were not compared with the control group.

Station-specific OSCE analyses were treated as secondary domain-level analyses. To account for the four station comparisons, a Bonferroni-adjusted two-sided threshold of *P* < 0.0125 was applied within this outcome family. Baseline characteristics were described using standardized effect sizes without significance testing. All 118 randomized participants had complete data for the reported outcomes and covariates; no imputation was required. The analysis hierarchy, number of tests, multiplicity approach, and exploratory or *post hoc* status of analyses are summarized in Supplementary Table S1. Assessment-form performance was explored descriptively by comparing total OSCE scores between Forms A and B. Because form assignment was completely determined by rotation batch, a form main effect could not be estimated independently in a model containing rotation-batch fixed effects. The between-form comparison was therefore interpreted descriptively rather than as a formal equivalence assessment. Potential heterogeneity of the intervention effect by assessment form was examined by adding a treatment-group-by-form interaction to the primary adjusted model; this analysis was exploratory. *Post hoc* sensitivity and exploratory analyses included additional adjustment of the primary model for age and gender; recalculation of the OSCE composite after excluding Station 3, which partially reused case contexts from Station 1; a more restrictive composite including only Stations 2 and 4, which did not share assessment cases with each other; analysis of Station 2 alone; analysis of the combined score for Stations 1, 3, and 4 after excluding the lesion-localization station; and treatment-group interactions with mean-centered digital literacy and mean-centered baseline academic performance. Alternative score outcomes were analyzed using the same adjusted linear-regression specification as the primary analysis. As an additional randomization-based sensitivity analysis, treatment labels were permuted within each rotation batch while preserving the observed number assigned to each group within that batch. For each of 20,000 permutations, the primary adjusted linear-regression model was refitted, and the observed absolute treatment coefficient was compared with its permutation-based null distribution. These analyses were outside the primary inferential framework. Because no unaltered, independently date-verifiable statistical analysis plan was retained, the timing of analyses described as planned before outcome examination cannot be independently confirmed; analyses developed after examination of the study data are identified as *post hoc*. The two-sided significance threshold for the primary endpoint was 0.05. Analyses were performed using IBM SPSS Statistics, version 27.0 (IBM Corp., Armonk, NY, USA). Figure 3 was generated from the reported model estimates using R version 4.5.2 (R Foundation for Statistical Computing, Vienna, Austria) and the ggplot2 package, version 4.0.3.

## 3 Results

### 3.1 Participant flow and baseline characteristics

All 118 eligible interns were randomized: 58 to AI-supported BOPPPS-CBL and 60 to conventional BOPPPS-CBL. All participants completed the primary OSCE and were included in the intention-to-treat analysis (Figure 1). Most baseline characteristics were closely balanced between groups. Age showed a modest standardized difference, with the AI-supported group being slightly younger (21.47 ± 0.86 vs. 21.73 ± 0.55 years; Cohen's *d* = −0.37). Baseline characteristics are presented in Table 1.

**Figure 1:**
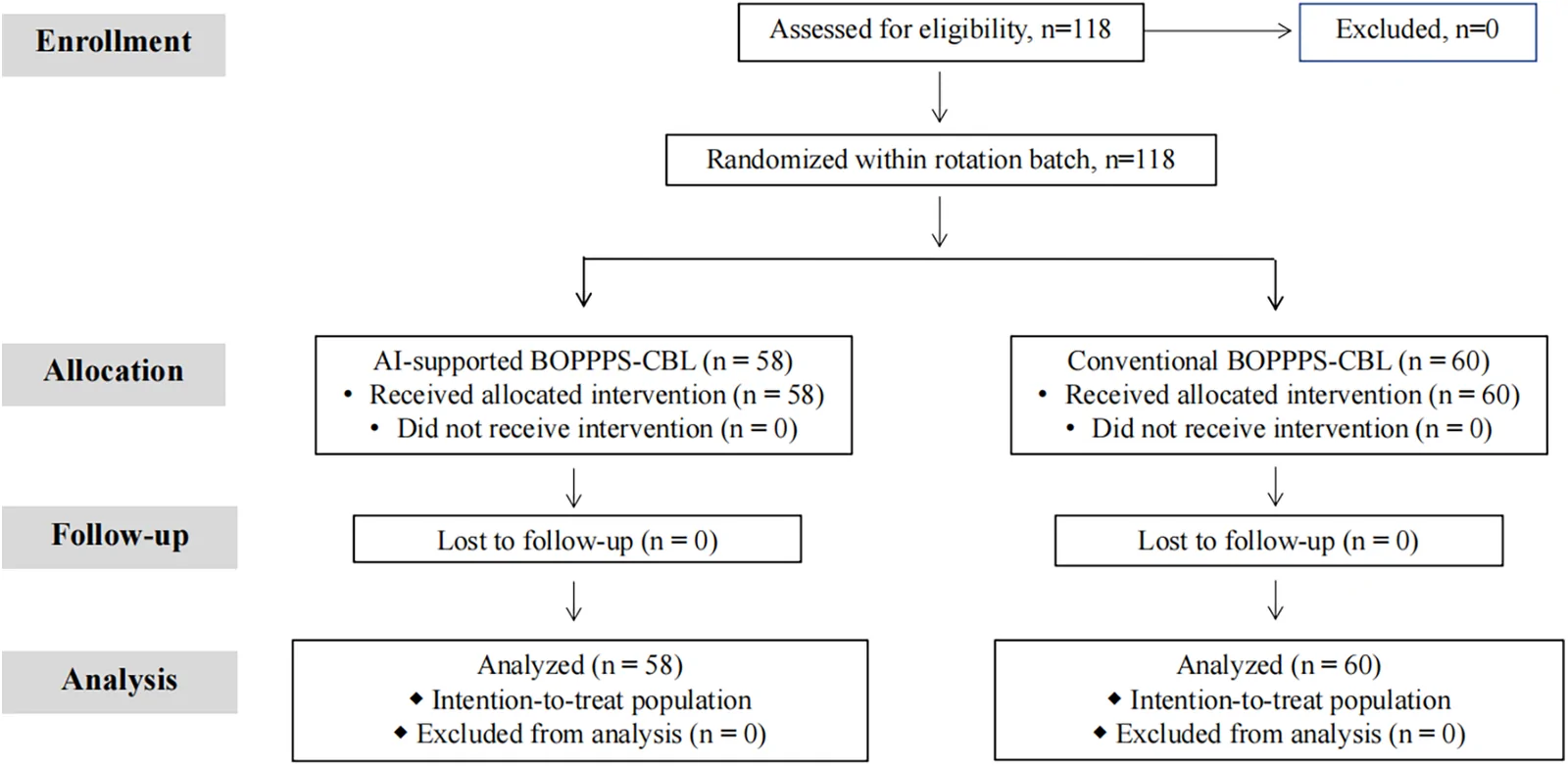
Participant flow through the batch-stratified randomized educational trial. A total of 118 eligible interns from 12 consecutive monthly rotation batches were individually randomized within batch to AI-supported BOPPPS-CBL (*n* = 58) or conventional BOPPPS-CBL (*n* = 60). All participants received the allocated intervention, completed the post-rotation assessments, and were included in the intention-to-treat analysis. No participants were lost to follow-up or excluded from analysis. AI, artificial intelligence; BOPPPS,Bridge-in, Objectives, Pre-assessment, Participatory Learning, Post-assessment, and Summary; CBL, case-based learning.

**Table 1:** Baseline characteristics of the randomized groups.

Variable	AI-BOPPPS (*n* = 58)	BOPPPS (*n* = 60)	Standardized effect size
Gender, *n* (%)			Cramer's V = 0.05
Male	26 (44.8)	30 (50.0)	
Female	32 (55.2)	30 (50.0)	
Age, years	21.47 ± 0.86	21.73 ± 0.55	Cohen's d = −0.37
Baseline clinically relevant academic performance score	82.22 ± 3.13	82.18 ± 2.92	Cohen's d = 0.01
Digital literacy score	3.49 ± 0.94	3.53 ± 0.93	Cohen's d = −0.05
Generative-AI use frequency, *n* (%)			Cramer's V = 0.03
Never	19 (32.8)	20 (33.3)	
Occasional: <1 time/week	21 (36.2)	20 (33.3)	
Frequent: ≥1 time/week	18 (31.0)	20 (33.3)	

Continuous variables are presented as mean ± SD and categorical variables as *n* (%). Baseline characteristics are described without significance testing. Standardized effect sizes are Cohen's d for continuous variables and Cramer's V for categorical variables. Baseline clinically relevant academic performance was calculated from the Diagnostics and Neurology course scores obtained in the semester immediately preceding the clinical internship. The two scores were combined using a credit-weighted mean.

### 3.2 Attendance, intervention exposure, and contamination

Attendance was complete in both groups: all 348 scheduled participant-session attendances in the AI-supported group and all 360 in the control group were recorded, corresponding to 100% attendance. These attendance counts document participation in the scheduled sessions but do not establish minutes spent in individual learning activities. All 58 intervention participants used the AI-supported approach during the rotation (AI-use coverage, 100%). No control participant reported study-related generative-AI use during the six modules (0/60).

### 3.3 OSCE score characteristics and inter-rater reliability

The locally developed OSCE underwent expert content review by five neurologists and feasibility pilot testing in 15 learners before study implementation; these procedures were not interpreted as formal psychometric validation. In the randomized sample, the mean total score was 79.20 ± 7.93, the median was 79.00 (IQR, 74.25–84.00), and the observed range was 60–96. No participant obtained the minimum or maximum possible total score. Station-level ceiling proportions ranged from 3.4% to 11.0%, and no station showed a floor effect. Inter-rater reliability for the total OSCE score, estimated using a two-way random-effects, absolute-agreement intraclass correlation coefficient for the mean of two assessors (ICC[2,2]), was 0.82. For station-level ratings pooled across all four stations, ICC[2,2] was 0.85. Separate station-specific ICCs and variance components were not estimated. No station assessment met the scoring-protocol threshold (0 of 472 station assessments). Forms A and B were each administered to 59 participants. Mean total OSCE scores were 79.41 ± 9.03 for Form A and 79.00 ± 6.74 for Form B, corresponding to a descriptive mean difference of 0.41 points (95% CI, −2.50 to 3.31; *P* = 0.782). Because form assignment was determined by alternating rotation batches, this comparison was descriptive and did not establish equivalence between the two forms. In an exploratory batch-adjusted interaction model, the treatment-group-by-Form B interaction estimate was −1.63 points (95% CI, −7.29 to 4.02; *P* = 0.568). Internal consistency across the four intentionally heterogeneous stations was low (Cronbach's *α* = 0.24), indicating limited covariance across station scores and limiting interpretation of the total as a unitary construct (Supplementary Table S4).

### 3.4 Primary outcome: simulated neurological-emergency assessment performance

The unadjusted mean OSCE total score was 82.07 ± 7.45 in the AI-supported group and 76.43 ± 7.43 in the control group. In the primary adjusted analysis, participants assigned to the AI-supported instructional package scored 5.67 points higher on the 100-point simulated assessment than those assigned to conventional BOPPPS-CBL (95% CI, 2.86–8.49; *P* < 0.001). The corresponding adjusted Cohen's d was 0.74 (Table 2). No minimal important difference or competency threshold had been prespecified for this locally developed assessment.

**Table 2:** Principal and domain-specific OSCE outcomes.

Outcome	AI-BOPPPS (*n* = 58) Mean ± SD	BOPPPS (*n* = 60) Mean ± SD	Adjusted mean difference (95% CI)	*P* value	Adjusted Cohen's d
OSCE total score	82.07 ± 7.45	76.43 ± 7.43	5.67 (2.86 to 8.49)	<0.001	0.74
Station 1: red-flag recognition	18.00 ± 4.10	16.67 ± 4.15	1.29 (-0.26 to 2.84)	0.101	0.30
Station 2: lesion localization	21.93 ± 2.99	19.40 ± 4.10	2.58 (1.25 to 3.91)	<0.001	0.71
Station 3: disposition and referral	20.74 ± 3.52	19.77 ± 3.09	0.99 (-0.27 to 2.26)	0.122	0.29
Station 4: examination integration	21.40 ± 2.26	20.60 ± 3.47	0.81 (-0.26 to 1.88)	0.137	0.28

Group values are unadjusted mean ± SD. Adjusted mean differences were estimated using linear regression with rotation-batch fixed effects and adjustment for baseline academic performance and digital literacy. Adjusted Cohen's d was calculated using the model residual SD. Alpha was 0.05 for the OSCE total and 0.0125 for each station-specific comparison.

### 3.5 Station-specific performance on the simulated assessment

Among the four station-specific analyses, the largest adjusted between-group estimate was observed for lesion localization (Station 2): 2.58 points on the 25-point station score (95% CI, 1.25–3.91; *P* < 0.001; adjusted Cohen's *d* = 0.71), meeting the Bonferroni-adjusted threshold of 0.0125. A Station 2-specific ICC was not estimated, and the pooled station-level ICC does not establish reliability for the lesion-localization station. Estimates were smaller for red-flag recognition (1.29 points; 95% CI, −0.26 to 2.84; *P* = 0.101), disposition and referral (0.99 points; 95% CI, −0.27 to 2.26; *P* = 0.122), and examination integration (0.81 points; 95% CI, −0.26 to 1.88; *P* = 0.137), with all three confidence intervals including zero (Table 2). Station 3 comprised two vignettes that reused case contexts previously encountered in Station 1 and contributed a maximum of 25 points to the 100-point OSCE total. Although Station 3 assessed disposition, immediate action, and timing rather than the red-flag-recognition construct assessed in Station 1, the extent to which prior case recognition influenced Station 3 performance could not be isolated. *Post hoc* sensitivity analyses therefore examined alternative score specifications. After excluding Station 3, the adjusted difference for the 75-point composite of Stations 1, 2, and 4 was 4.68 points (95% CI, 2.23–7.13; *P* < 0.001; adjusted *d* = 0.70). For the 50-point composite containing only Stations 2 and 4, which did not share assessment cases with each other, the adjusted difference was 3.39 points (95% CI, 1.57–5.21; *P* < 0.001; adjusted *d* = 0.68). The Station 2-only estimate was 2.58 points (95% CI, 1.25–3.91; *P* < 0.001; adjusted *d* = 0.71). These *post hoc* analyses yielded standardized estimates of similar magnitude to the primary four-station analysis but do not eliminate the possibility of cueing or carry-over from reused cases. In a separate *post hoc* analysis excluding Station 2, the adjusted difference for Stations 1, 3, and 4 was 3.09 points (95% CI, 0.66–5.53; *P* = 0.013). Additional adjustment for age and gender yielded an adjusted total-score difference of 5.46 points (95% CI, 2.63–8.29; *P* < 0.001). The exploratory treatment-group-by-digital-literacy interaction was not clearly different from zero (interaction estimate, 1.17 points per one-point increase in digital literacy; 95% CI, −1.92 to 4.27; *P* = 0.454). Similarly, the *post hoc* treatment-group-by-baseline-academic-performance interaction was 0.72 points per one-point increase in baseline academic performance (95% CI, −0.22 to 1.66; *P* = 0.132), providing no clear evidence that the estimated intervention effect varied with baseline academic performance (Supplementary Table S5).

### 3.6 End-of-rotation written examination

The unadjusted written-examination total score was 76.60 ± 11.85 in the AI-supported group and 73.38 ± 10.20 in the control group. The adjusted between-group difference was 3.22 points on the 100-point examination (95% CI, −0.97 to 7.41; *P* = 0.131; adjusted Cohen's *d* = 0.28). Adjusted differences were 0.26 points for the 50-point MCQ component (95% CI, −1.83 to 2.35; *P* = 0.807; adjusted *d* = 0.05) and 2.96 points for the 50-point KFQ component (95% CI, −0.49 to 6.41; *P* = 0.092; adjusted *d* = 0.31) (Table 3). These secondary outcome estimates were imprecise, and all confidence intervals included zero.

**Table 3:** Post-rotation written-examination outcomes.

Outcome	AI-BOPPPS (*n* = 58) Mean ± SD	BOPPPS (*n* = 60) Mean ± SD	Adjusted mean difference (95% CI)	*P* value	Adjusted Cohen's d
MCQ score (0–50)	39.10 ± 5.53	38.88 ± 5.56	0.26 (-1.83 to 2.35)	0.807	0.05
KFQ score (0–50)	37.50 ± 10.11	34.50 ± 7.97	2.96 (-0.49 to 6.41)	0.092	0.31
Total score (0–100)	76.60 ± 11.85	73.38 ± 10.20	3.22 (-0.97 to 7.41)	0.131	0.28

MCQ, multiple-choice question; KFQ, key-feature question. Group values are unadjusted mean ± SD; adjusted estimates were obtained using the same regression model as in Table 2.

### 3.7 Exploratory post-rotation questionnaire outcomes

Internal consistency was low for the study-developed self-regulated-learning (Cronbach's *α* = 0.37) and satisfaction (*α* = 0.59) item sets and was negative for the perceived-burden item set (*α* = −0.17). Accordingly, no composite scores were interpreted, and the 14 individual items were treated as exploratory learner-reported outcomes (Figure 3; Supplementary Tables S6, S7). All 118 participants contributed responses to these analyses. Observed response-category distributions are reported in Supplementary Table S6.

**Figure 2:**
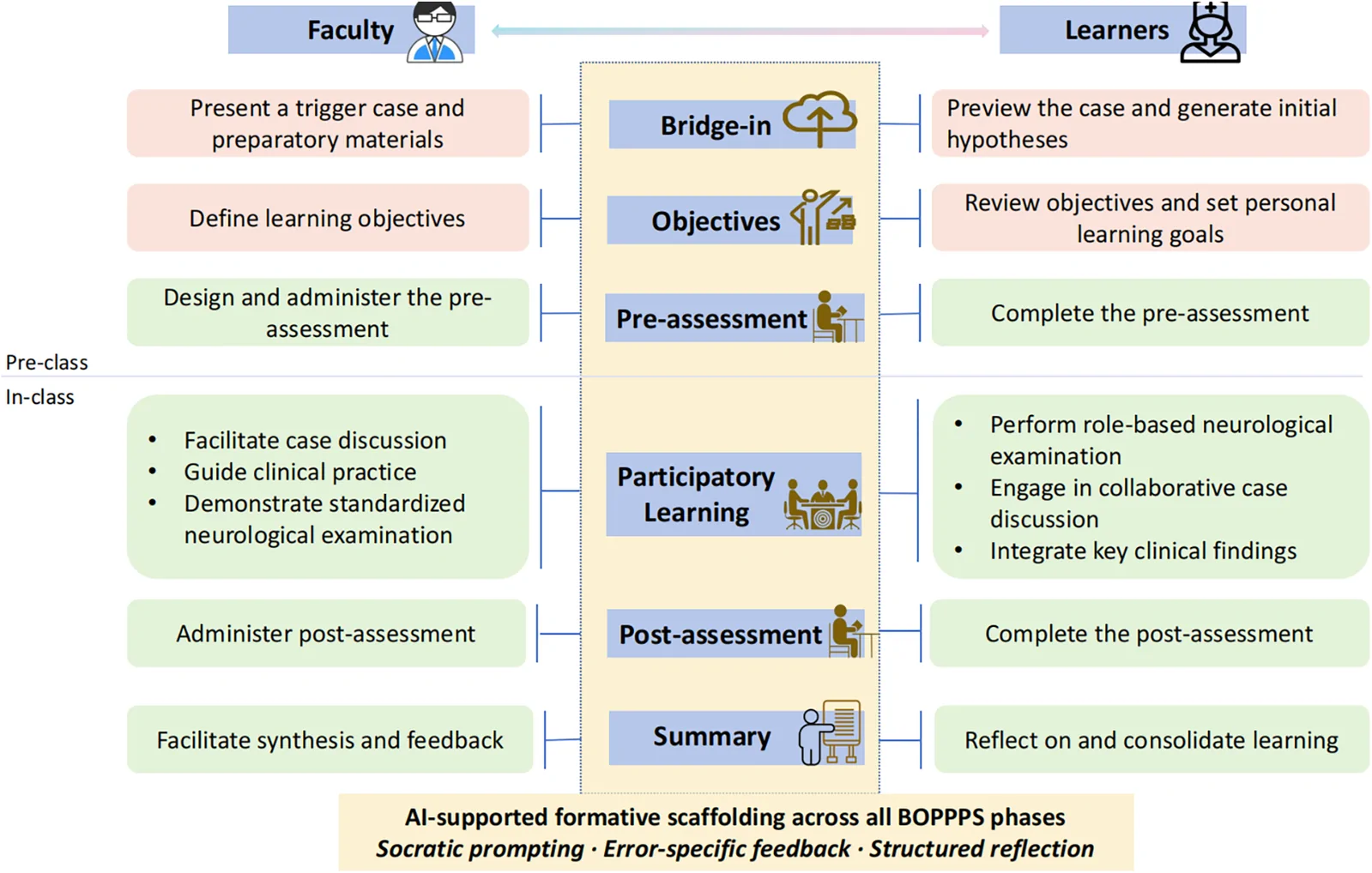
Operational framework of the AI-supported BOPPPS-CBL intervention. The central column shows the six sequential BOPPPS phases, with corresponding faculty activities on the left and learner activities on the right. The horizontal divider distinguishes the planned pre-class and in-class components. Faculty and learner activities were shared across both study groups; the AI-supported formative-scaffolding layer was added only in the intervention group. This layer operated across all six phases through Socratic prompting, error-specific feedback, and structured reflection. Learners were required to provide an initial response before receiving AI feedback, and the AI did not grade performance, replace teacher judgment, or contribute to summative assessment. Teachers monitored the learning process and corrected inaccurate or unsafe outputs when necessary. Detailed prompt templates and implementation procedures are provided in Supplementary Appendix S1 and Supplementary Table 3. AI, artificial intelligence; BOPPPS,Bridge-in, Objectives, Pre-assessment, Participatory Learning, Post-assessment, and Summary; CBL, case-based learning.

**Figure 3:**
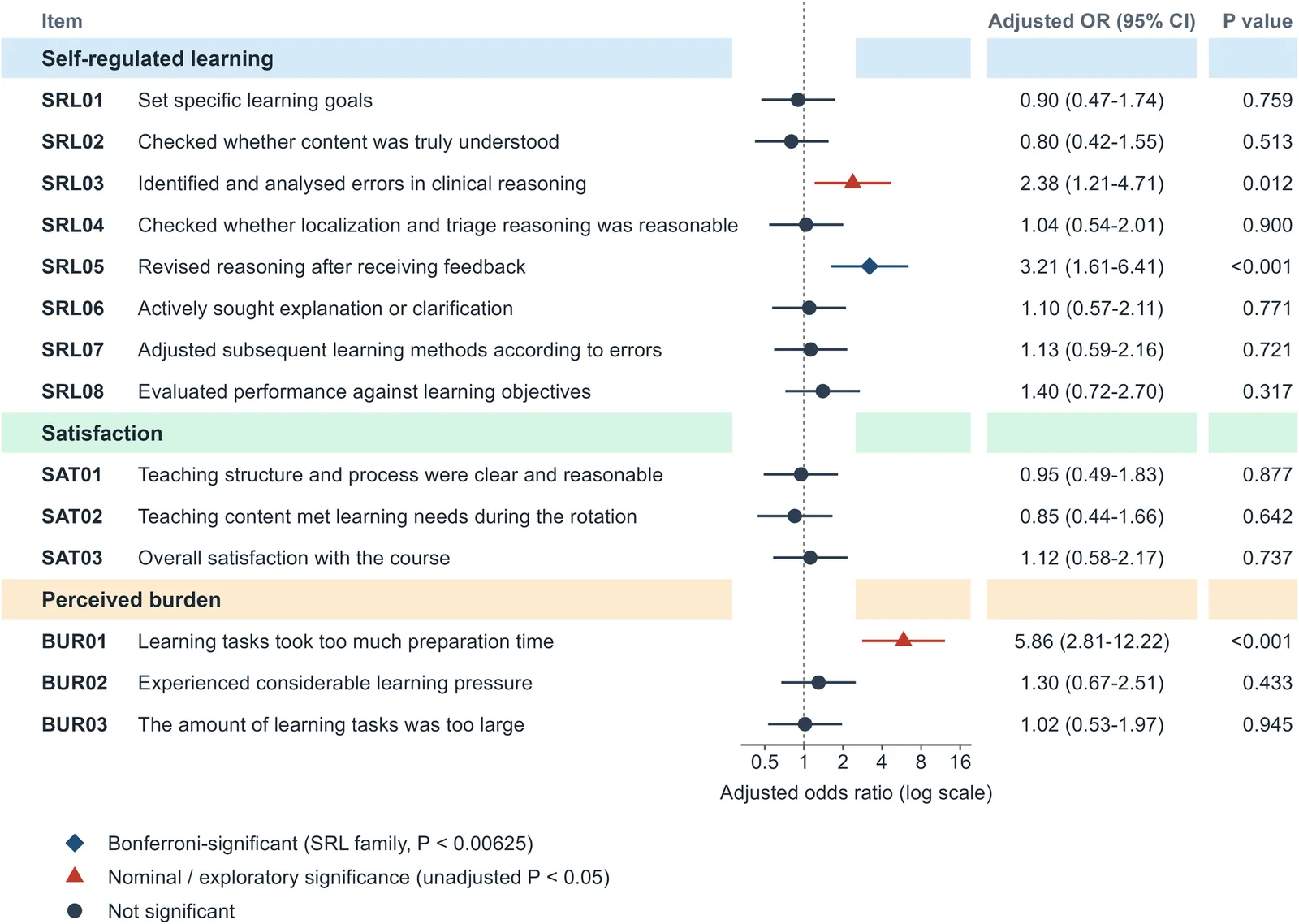
Item-level adjusted odds ratios for post-rotation questionnaire outcomes comparing AI-supported BOPPPS-CBL with conventional BOPPPS-CBL. Points show adjusted odds ratios (OR) and horizontal bars show 95% confidence intervals on a logarithmic scale; the dashed line marks OR=1. Estimates were obtained from proportional-odds logistic models adjusted for rotation batch, baseline academic performance, and digital literacy. OR > 1 indicates greater odds of selecting a higher Likert category in the AI-supported arm. For BUR01, this corresponds to greater perceived preparation-time burden and should not be interpreted as a favorable outcome. Diamonds denote items that met the Bonferroni-adjusted threshold for the eight self-regulated-learning items (*P* < 0.00625). Triangles denote items with an unadjusted *P* < 0.05 only. Filled circles denote items that were not statistically significant. Satisfaction and perceived-burden items were treated as exploratory and were not adjusted for multiplicity.

In the original five-category proportional-odds models, participants assigned to the AI-supported instructional package more often selected higher response categories for revision of reasoning after feedback (SRL05: adjusted OR, 3.21; 95% CI, 1.61–6.41; *P* < 0.001). This result met the Bonferroni-adjusted threshold of *P* < 0.00625 for the eight self-regulated-learning items. For identifying and analysing errors in clinical reasoning (SRL03), the adjusted OR was 2.38 (95% CI, 1.21–4.71; *P* = 0.012), which did not meet the adjusted threshold in the original model. The remaining self-regulated-learning items showed no clear between-group differences. None of the three satisfaction items showed a clear between-group difference. For perceived burden, participants assigned to the AI-supported package more often selected higher response categories for excessive preparation time (BUR01: adjusted OR, 5.86; 95% CI, 2.81–12.22; *P* < 0.001). Estimates for learning pressure (BUR02: adjusted OR, 1.30; 95% CI, 0.67–2.51; *P* = 0.433) and excessive task volume (BUR03: adjusted OR, 1.02; 95% CI, 0.53–1.97; *P* = 0.945) were imprecise and included the null value.

The proportional-odds assumption was not rejected for any of the 14 original five-category models. In sensitivity analyses that collapsed responses into three ordered categories, the estimates for SRL05 and BUR01 remained similar in direction and magnitude (SRL05: adjusted OR, 3.60; 95% CI, 1.69–7.68; *P* < 0.001; BUR01: adjusted OR, 6.71; 95% CI, 3.13–14.38; *P* < 0.001). The collapsed-category estimate for SRL03 was stronger (*P* = 0.002), indicating some sensitivity to response-category specification; this item was therefore retained as an exploratory finding rather than interpreted as a confirmatory effect. For SAT02, the collapsed-category model showed evidence against the proportional-odds assumption (parallel-lines *P* = 0.013). A supplementary binary logistic-regression sensitivity analysis comparing agree/strongly agree with all other responses remained null (adjusted OR, 0.79; 95% CI, 0.36–1.73; *P* = 0.559). No other collapsed-category model showed clear evidence against the proportional-odds assumption (Supplementary Table S7).

### 3.8 AI-assisted learning experience

Among the 58 participants in the AI-supported group, the exploratory item-grouping means ranged from 3.72 to 3.78 on the 5-point scale, with medians of 4.00. Internal consistency ranged from 0.51 for trust in AI feedback to 0.70 for perceived usefulness (Table 4). Item-level counts and percentages across all five response categories for each of the 12 intervention-only AI-experience items are provided in Supplementary Table S8 (*n* = 58 per item). Because most groupings had limited internal consistency and were based on study-developed items, these findings are presented as descriptive summaries rather than validated construct-level estimates.

**Table 4:** Exploratory descriptive summaries of AI-assisted learning experience in the AI-BOPPPS group (*n* = 58).

Exploratory item grouping	No. of items	Mean ± SD	Median (IQR)	Cronbach's *α*
Perceived usefulness	3	3.78 ± 0.85	4.00 (3.67 to 4.33)	0.70
Perceived ease of use	3	3.76 ± 0.83	4.00 (3.67 to 4.33)	0.62
Trust in AI feedback	2	3.74 ± 0.91	4.00 (3.50 to 4.38)	0.51
Possible reliance on AI prompting	2	3.72 ± 0.97	4.00 (3.50 to 4.50)	0.68
Future use intention	2	3.75 ± 0.93	4.00 (3.50 to 4.50)	0.59

Scores range from 1 to 5. Higher values indicate stronger endorsement of the item grouping. For possible reliance on AI prompting, higher scores indicate greater self-reported reliance risk. These study-developed groupings were summarized descriptively and were not interpreted as validated constructs or compared with the control group.

## 4 Discussion

### 4.1 Principal findings

In this batch-stratified randomized educational study, participants assigned to the AI-supported BOPPPS-CBL instructional package scored 5.67 points higher on the 100-point locally developed simulated neurological-emergency assessment than those assigned to conventional BOPPPS-CBL after adjustment for rotation batch, baseline academic performance, and digital literacy. The largest station-specific estimate was observed for lesion localization, but a Station 2-specific ICC was not available; the pooled station-level ICC cannot establish reliability for this station. *Post hoc* analyses excluding Station 3, which reused case contexts from Station 1, and restricting the outcome to Stations 2 and 4 yielded standardized estimates of similar magnitude to the primary analysis. These sensitivity analyses reduce concern that the observed difference was driven entirely by the shared-case station, but they do not eliminate possible cueing or carry-over and were not prospectively specified. Estimates for the written-examination total, MCQ, and KFQ components were smaller and imprecise, with confidence intervals including zero. The study-developed questionnaire item sets showed low or negative internal consistency and therefore did not support construct-level conclusions about self-regulated learning, satisfaction, or perceived burden. At the item level, participants assigned to the AI-supported package more often selected higher response categories for revising reasoning after feedback and for excessive preparation time. These learner-reported findings should be considered exploratory.

### 4.2 Comparison with prior work

The present study extends our earlier randomized educational study of EBM-integrated BOPPPS in neurology clerkships for three-year junior-college medical clerks (18). The earlier study evaluated evidence-based-medicine training, whereas the present study evaluated a faculty-supervised DeepSeek scaffold embedded within BOPPPS-CBL among medical interns. The participant cohorts, assessment instruments, cases, teaching faculty, standardized patients, and assessors were separate. More broadly, previous studies and reviews have evaluated BOPPPS, case-based learning, and generative-AI-supported feedback in health-professions education (22–24). The larger estimate for the simulated assessment than for the written examination is compatible with the possibility that the instructional package supported the organization, articulation, and revision of reasoning processes more than factual knowledge acquisition alone. This interpretation remains tentative because treatment effects were not formally compared across outcome domains.

### 4.3 Potential mechanisms and interpretation of the findings

Individualized Socratic prompting, error-focused feedback, structured reflection, and faculty correction may have made otherwise implicit reasoning steps more visible and amenable to monitoring and revision (17, 25). The lesion-localization estimate was the only station-specific result meeting the multiplicity-adjusted threshold and was most consistent with the intervention's required localization maps and stepwise prompting. As a secondary domain analysis, however, it should not be treated as independent confirmatory evidence. The learner-reported feedback-revision item was also compatible with the proposed mechanism. However, the intervention was delivered as a bundled instructional package. It incorporated AI-mediated prompts, structured reflection, faculty oversight, and potentially greater learner effort than the comparator. The study therefore cannot isolate the contribution of AI-generated feedback from that of additional deliberate practice, feedback exposure, or time on task. In addition, the self-regulated-learning items were administered only after the rotation, had limited internal consistency, and were not evaluated in a mediation analysis. The questionnaire findings should therefore be regarded as exploratory observations rather than evidence that changes in self-regulation mediated the difference in simulated assessment scores.

### 4.4 Interpretation of learner-reported self-regulated learning

The self-regulated-learning findings should be interpreted at the item level rather than as evidence of a generalized change in self-regulation. In the original five-category analysis, only the item concerning revision of reasoning after feedback met the multiplicity-adjusted threshold. The error-analysis item showed a directionally compatible but less certain pattern and crossed the adjusted threshold only after response categories were collapsed in a sensitivity analysis, indicating some dependence on model specification. Goal setting, monitoring, help seeking, strategy adjustment, and self-evaluation showed no clear between-group differences. This pattern is compatible with models in which feedback can support monitoring and strategy revision, but the study-developed measure, its low internal consistency, and the sensitivity of some item-level estimates to response-category specification do not permit a broader conclusion about metacognitive development (25).

### 4.5 Acceptability, AI reliance, and implementation burden

Exploratory descriptive summaries within the intervention group indicated generally favorable responses regarding usefulness, ease of use, trust, and future use, together with some concern about reliance on AI prompting. Because these study-developed item groupings had limited internal consistency, they should not be interpreted as validated measures of acceptability or AI reliance. Over-reliance, inaccurate output, and the continuing need for educator oversight remain important considerations in generative-AI-supported medical education (26). Participants assigned to the AI-supported package more often reported that the learning activities required excessive preparation time. However, the study did not prospectively record time on task in minutes or hours, nor did it separately quantify time devoted to AI dialogue, reflection artifacts, or faculty interaction. The magnitude and source of the additional workload therefore cannot be determined from the present data. Future implementation studies should prospectively record activity-specific learner and faculty time. Requiring an independent initial response before AI feedback may remain useful for limiting uncritical reliance, but its effectiveness should be evaluated directly rather than inferred from the present study (27).

### 4.6 Public-health and educational implications

The potential public-health relevance of this approach lies in health-professions education rather than demonstrated patient-care effects. County hospitals and township health centers manage large patient volumes (4), and supervised AI scaffolding may warrant further evaluation in settings where individualized faculty feedback is difficult to provide. Wider implementation would require prospective assessment of costs, connectivity, privacy, equity, and faculty workload because digital education may reproduce or widen existing disparities (28, 29). Any such implementation should preserve independent clinical judgment and maintain faculty oversight and accountability (26). The present findings are limited to performance on a simulated educational assessment and do not demonstrate faster referral, safer workplace decisions, improved patient safety, reduced disability, or other patient outcomes.

### 4.7 Limitations

This study has several limitations. First, it was not prospectively registered, and no public trial registration identifier or registry URL exists. Internal working drafts were revised in place, and no original, independently date-verifiable protocol or statistical analysis plan was retained. The chronology of outcome prioritization and analytical decisions therefore cannot be independently verified, although analyses developed after examination of the study data are identified as *post hoc*. Second, the intervention was a bundled instructional package incorporating AI-mediated prompting, structured reflection, faculty oversight, and learner practice. The study cannot separate the contribution of AI-generated feedback from differences in deliberate practice, feedback exposure, learner effort, or time on task. Although participants reported greater preparation-time burden, minutes spent in preparation outside scheduled sessions, AI dialogue, reflection, and activity-specific faculty contact were not recorded; the delivered activity-specific dose therefore remains uncertain. Third, the study was conducted at one hospital and one college. Learners and faculty could not be blinded, creating the possibility of expectation, performance, and differential-attention effects. OSCE assessors were blinded, but whether standardized patients or assessors could ever infer allocation was not formally assessed. Fourth, contamination monitoring in the control group relied on participant self-report. Although no control participant reported study-related generative-AI use during the intervention, unreported use, use of other AI tools, or cross-group sharing cannot be excluded. Fifth, session-level implementation records were incomplete. Model version and optional settings, prompt volume, completion of individual scaffold stages, premature answer disclosure, faculty corrections, and session-level unsafe or inaccurate outputs were not logged systematically. Intervention fidelity and model drift therefore could not be quantified retrospectively. Sixth, the primary outcome was a locally developed simulated performance measure rather than an independently validated measure of clinical competence. Expert review and feasibility pilot testing supported content relevance and administration, but criterion validity, known-groups validity, test-retest reliability, generalizability analysis, and external validation were not established. Separate station-specific ICCs and variance components were not estimated, limiting the reliability evidence for domain-level findings such as lesion localization. Internal consistency across the four station scores was low (Cronbach's *α* = 0.24). Although heterogeneous station content was intentional, this limits interpretation of the total score as a unitary measure of neurological-emergency reasoning. Seventh, Station 3 reused two case contexts previously encountered in Station 1, creating the possibility of recognition, cueing, or carry-over. *Post hoc* analyses excluding Station 3 and restricting the outcome to nonshared-case stations yielded estimates of similar magnitude, but these analyses cannot eliminate this potential bias. In addition, Forms A and B were determined by alternating rotation batches and were therefore confounded with batch and time. Their descriptive score similarity does not establish statistical equivalence. Eighth, the study-developed self-regulated-learning, satisfaction, and perceived-burden item sets had low or negative internal consistency. Their item-level analyses should therefore be considered exploratory and are vulnerable to sparse response categories and multiplicity. The baseline digital-literacy measure was also study-developed; only its composite score was retained in the analytic dataset, preventing retrospective assessment of internal consistency. Finally, the study assessed outcomes only at the end of the rotation. It did not evaluate retention, workplace transfer, referral behavior, patient safety, or patient outcomes. A modest baseline age imbalance was also present, although a *post hoc* model with additional adjustment for age and gender produced a similar primary estimate.

### 4.8 Future directions

The current simulated assessment primarily addresses the “shows how” level rather than workplace “does” performance in Miller's framework (30). Future multicenter studies should therefore use independently or externally validated assessment frameworks, assess retention at 3–6 months, and include workplace-based outcomes such as supervised case logs, referral decisions, and supervisor ratings. A three-arm design could help separate the contribution of AI from additional feedback and practice time by comparing conventional BOPPPS-CBL, time- and feedback-matched non-AI structured scaffolding, and AI-supported scaffolding. Future trials should prospectively register the protocol and statistical analysis plan, predefine outcome hierarchies and clinically or educationally meaningful score differences where feasible, and collect detailed intervention-fidelity data. These records should include learner time on task, faculty contact time, prompt volume, model/version information, correction events, and inaccurate or unsafe outputs. Better-validated measures of self-regulated learning, digital literacy, acceptability, and implementation burden are also needed. Longer-term work should evaluate whether any improvement observed in simulated assessments transfers to real clinical settings before patient-care or population-health benefit is inferred.

## 5 Conclusion

Among junior-college medical interns, assignment to a faculty-supervised AI-supported BOPPPS-CBL instructional package was associated with higher scores on a locally developed simulated neurological-emergency assessment than assignment to conventional BOPPPS-CBL. The largest station-specific estimate was observed for lesion localization, although the absence of a Station 2-specific reliability estimate limits interpretation of this secondary finding. *Post hoc* sensitivity analyses excluding the station containing reused case contexts yielded estimates of similar magnitude, but these analyses do not eliminate potential cueing or carry-over. Written-examination estimates were smaller and imprecise, while exploratory learner-reported findings suggested greater perceived preparation-time burden. Multicenter studies using independent assessment cases, longer-term follow-up, and workplace-based outcomes are needed before effects on clinical performance, patient care, or population health can be inferred.

## Data Availability

The raw data supporting the conclusions of this article will be made available by the authors, without undue reservation.
