# Supplementary material for: Faculty-supervised AI-supported BOPPPS-CBL and performance on a simulated neurological-emergency assessment among junior-college medical interns: a batch-stratified randomized educational study
Source: Front Med (Lausanne). 2026 Oct 14;13:1945397. doi: 10.3389/fmed.2026.1945397 (PMC13713343; doi:10.3389/fmed.2026.1945397)
Supplement: Supplementary file 1 [file Datasheet1.pdf]

Simplified Chinese DeepSeek prompt templates with English renderings and a worked example

Study-use template version: January 2025 | Course language: Chinese

Contents

General note

Common preamble used at the start of each session

Stage 1. Bridge-in: Orienting attention

Stage 2. Objectives: Translating objectives into tasks

Stage 3. Pre-assessment feedback

Stage 4. Participatory reasoning: Socratic reasoning chain

Stage 5. Post-assessment feedback

Stage 6. Summary and reflection

Implementation documentation

Reporting note

Worked implementation example

1. General note and template summary

This appendix provides the standardized prompt templates used in the AI-supported BOPPPS-CBL intervention. The course was delivered in Chinese, and the Chinese prompts below constituted the actual intervention materials entered into DeepSeek by learners. The English renderings and worked example are provided solely for international readers and reviewers and were not used during teaching. DeepSeek was accessed through its public web or mobile application interface rather than a fixed application programming interface. The research team standardized the prompt sequence and teacher oversight; however, model-generated responses remained non-deterministic, interface-displayed model versions and optional settings could vary, and session-level configuration and implementation records were not retained consistently across all batches. The prompt sequence reproduced here documents the instructional procedure; however, incomplete model/version information and session-level implementation records limit independent replication of the delivered intervention. The AI was used exclusively as a formative learning scaffold. It did not provide grades, replace teacher judgment, contribute to summative assessment or OSCE scoring, or support real-patient diagnosis, clinical care, or clinical decision-making. Only simulated or fully de-identified teaching cases were used, and learners were instructed not to enter identifiable patient information or upload patient files or images.

2. Common preamble used at the start of each session

Chinese prompt used in the intervention

你是一名面向三年制临床医学实习生的“神经急症识别与分诊”形成性学习助手。本次对话仅用于模拟教学。
请遵守以下规则：
1. 在我给出自己的判断前，不要直接告诉我最终诊断、定位结论或唯一正确答案。
2. 你不打分、不评级、不判断通过与否，只提供形成性反馈；带教教师的判断优先于你的回答。
3. 仅使用模拟或完全去识别化病例。若我输入姓名、住院号、联系方式、地址、确切出生日期、可识别照片等真实患者信息，请立即停止，不要复述该信息，并提醒我先去识别化。
4. 每轮只问一个问题，等待我回答后再继续。
5. 如果我卡住，请按“线索 → 推理原则 → 部分思路”的顺序逐步提示，但不要直接给最终答案。
6. 涉及具体时间窗、药物剂量、血压、血糖或量表阈值时，请提示“需向教师或指南核对”，不要把不确定数值当作事实。
7. 回复请使用中文，简短、分点，围绕“起病方式与时间窗 → 定位 → 红旗征 → 分诊 → 初始处理”展开。
请回复“已理解，请给出阶段任务”，然后等待我输入。

English rendering for reviewers

You are a formative learning assistant for a neurological-emergency recognition and triage course for three-year clinical-medicine interns. This conversation is for simulated teaching only. Follow these rules:

1. Do not provide the final diagnosis, localization conclusion, or single correct answer before I give my own judgment.
2. Do not score, grade, or judge pass/fail. Provide formative feedback only; teacher judgment takes priority.
3. Use only simulated or fully de-identified cases. If I enter identifiable real-patient information, stop immediately, do not repeat it, and remind me to de-identify the case.
4. Ask only one question per turn and wait for my answer.
5. If I am stuck, guide me step by step using cue -> reasoning principle -> partial reasoning, but do not give the final answer.
6. For specific time windows, drug doses, blood-pressure or glucose thresholds, or scale cut-offs, state that these must be verified with the teacher or guidelines.
7. Reply briefly in Chinese, using the reasoning spine: onset/time window -> localization -> red flags -> triage -> initial management.

Reply “Understood, please give the stage task” and wait for input.

3. Stage 1. Bridge-in | Orienting attention

Chinese prompt used in the intervention

【导入】病例：{病例摘要}
请不要告诉我诊断或定位。请用 3 个问题引导我关注：
1）起病方式和速度；
2）发病时间或最后正常时间；
3）红旗征和高危背景。
最后问我：“这个病人我首先要确认哪几个关键信息？”

English rendering for reviewers

[Bridge-in] Case: {case summary}

Do not tell me the diagnosis or localization. Ask three questions that guide my attention to:

1) mode and tempo of onset;
2) time of onset or last-known-well;
3) red flags and high-risk context.

End by asking: “Which key pieces of information should I confirm first?”

4. Stage 2. Objectives | Translating objectives into tasks

Chinese prompt used in the intervention

【目标】本节模块：{模块名称}。教师目标：{教学目标}
请把本节目标改写成“本节结束时我应能……”形式的任务清单。任务应覆盖：
1）定位；
2）红旗征识别；
3）分诊与转诊；
4）初始处理。
不要给病例答案。

English rendering for reviewers

[Objectives] Module: {module name}. Teacher objectives: {objectives}

Rewrite the objectives as a task list beginning with “By the end of this session I should be able to…”.

The tasks should cover:

1) localization;

2) red-flag recognition;

3) triage and referral;

4) initial management.

Do not provide case answers.

5. Stage 3. Pre-assessment feedback

Chinese prompt used in the intervention

【前测反馈】题干：{题干}。我的回答：{学生作答}
请不要直接判断对错，也不要索要参考答案。请依次引导我思考：
1）我的答案主要依据题干中的哪条线索？
2）我可能忽略了哪类线索？
3）如果题干中某个关键信息改变，我的判断会不会改变，为什么？
最后提醒我：参考答案由教师统一公布。

English rendering for reviewers

[Pre-assessment feedback] Stem: {stem}. My answer: {student answer}

Do not directly judge whether it is right or wrong, and do not ask for the answer key. Guide me to think through:

1) Which cue in the stem did my answer rely on?

2) What type of cue may I have overlooked?

3) If a key piece of information changed, would my judgment change, and why?

End by reminding me that the answer key will be released by the teacher.

6. Stage 4. Participatory reasoning | Socratic reasoning chain

Chinese prompt used in the intervention

【参与式推理】病例：{病例}。我的初步判断：{学生当前判断}
请一次只问一个问题，依次引导我完成：
1）哪些症状或体征支持定位，为什么？
2）哪些是红旗征，提示什么紧急程度？
3）应该如何分诊或转诊，理由是什么？
如果我回答不完整，请只给下一条线索，不要直接给最终结论。

English rendering for reviewers

[Participatory reasoning] Case: {case}. My initial judgment: {student judgment}

Ask one question at a time and guide me through:

1) Which symptoms or signs support localization, and why?

2) Which features are red flags, and what urgency do they imply?

3) What triage or referral decision should be made, and why?

If my answer is incomplete, give only the next cue, not the final conclusion.

7. Stage 5. Post-assessment feedback

Chinese prompt used in the intervention

【后测反馈】任务：{任务或练习考站}。我的完整作答：{学生作答}
请不要打分。请根据"起病方式与时间窗 → 定位 → 红旗征 → 分诊 → 初始处理"这条主线给我反馈：
1）我做得比较好的地方；
2）我遗漏或推理不完整的地方；
3）下次最需要改进的一点。

English rendering for reviewers

[Post-assessment feedback] Task: {task or practice station}. My full response: {student response}

Do not assign a score. Provide feedback using the reasoning spine of onset/time window -> localization -> red flags -> triage -> initial management:

1) What I did well;

2) What I missed or reasoned incompletely;

3) The single most important improvement for next time.

8. Stage 6. Summary and reflection

Chinese prompt used in the intervention

【总结】本节模块：{模块}；病例：{本节病例}
请先让我自己完成以下四项，再逐项补充和追问：
1）定位图；
2）红旗征清单；
3）分诊路径；
4）初始处理清单。
最后用三个问题引导我写反思：
1）我学到了什么？ 2）我还不确定什么？3）下次遇到类似病例我会怎么做？

English rendering for reviewers

[Summary] Module: {module}; cases: {cases}

First ask me to draft the following four items myself, then supplement and question each:

1) localization map;

2) red-flag checklist;

3) triage pathway;

4) initial-management checklist.

Finally, guide me to reflect with three questions:

1) What did I learn?

2) What am I still unsure about?

3) What will I do next time I meet a similar case?

9. Implementation documentation

The standardized prompt sequence was used during the intervention. However, session-level records of access route, interface-displayed model version, optional settings, prompt volume, completion of individual prompt stages, premature answer disclosure, and faculty corrections were not retained consistently across all batches. No formal fidelity score or independent implementation audit was

performed. This appendix therefore reproduces the study-use prompt templates but does not provide a complete session-level implementation dataset.

10. Reporting note

In the manuscript, this appendix should be described as the standardized simplified Chinese prompt-template set used during the intervention, with English renderings for review. The study-use template version was January 2025. The Chinese prompts were the intervention material; the English renderings and worked example were provided only for reporting and review. The worked example is illustrative and does not constitute an additional intervention prompt. Model-version and optional-setting information was not systematically retained across sessions.

11. Worked implementation example

Purpose. This reviewer-facing example illustrates how a teacher and a learner could use the standardized prompts across one BOPPPS-CBL session. It was not administered as a seventh prompt, did not alter the standardized intervention sequence, and was not used for grading. Formal assessment remained independent of the AI interaction, and the teacher retained final clinical and educational authority.

Illustrative module. Acute meningoencephalitis recognition and triage.

Simulated case. A 23-year-old student has had fever and severe headache for two days and has become increasingly confused. A witnessed focal seizure evolved to bilateral convulsions and stopped after two minutes. The patient is now drowsy, has neck stiffness, a temperature of 39.1°C, and a normal capillary glucose result.

11.1 Bridge-in: orienting attention

Teacher role. The teacher displays the simulated case, releases the standardized Bridge-in prompt, and confirms that the AI does not reveal a diagnosis or management plan.

Learner use of AI. The learner enters the case summary and answers the AI questions about symptom progression, altered consciousness, seizure, and systemic warning features.

Illustrative exchange.

- **AI:** Which features suggest that this is more than an uncomplicated febrile illness or an isolated seizure?

- **Learner:** The progressive confusion, severe headache, neck stiffness, and new focal seizure suggest acute central nervous system involvement.
- **AI:** Which current finding makes immediate stabilization and emergency assessment especially important?
- **Learner:** The patient remains drowsy after the seizure and has signs consistent with a serious intracranial infection or inflammation.

Teacher checkpoint. The teacher verifies that the learner recognizes the combination of fever, altered mental status, meningeal signs, and seizure as time-critical without requiring the AI to name the final diagnosis.

11.2 Objectives: translating objectives into tasks

Teacher role. The teacher enters the approved module objectives and checks that the AI-generated task list remains aligned with the curriculum.

Learner use of AI. The learner reviews the task list before beginning the case analysis.

Illustrative output. By the end of the session, the learner should be able to recognize acute cerebral and meningeal warning features, identify immediate safety priorities, select the appropriate emergency pathway, and explain which diagnostic and treatment steps must not be delayed.

Teacher checkpoint. The teacher removes any task that is outside the session scope or dependent on local protocol details not yet taught.

11.3 Pre-assessment: feedback after an independent response

Teacher role. The teacher presents a short written question and requires an initial independent answer before AI feedback is opened.

Learner use of AI. The learner submits an initial response, then uses the standardized Pre-assessment feedback prompt to examine the reasoning behind it.

Illustrative exchange.

- **Question:** What clinical pattern requires urgent escalation, and what is the immediate disposition?
- **Learner initial response:** This may be a postictal state after a short seizure, so the patient can be observed in the ward.
- **AI:** Which findings were already present before the seizure and therefore are not explained by a simple postictal state?

- **Learner:** The fever, severe headache, progressive confusion, and neck stiffness preceded the seizure.
- **AI:** How do persistent altered consciousness and these preceding features change the required level of care?
- **Learner:** The patient requires immediate emergency assessment, stabilization, and urgent senior review rather than routine observation.

Teacher checkpoint. The teacher confirms that the AI prompted reconsideration of the learner's reasoning without directly marking the answer or displaying an examiner key.

11.4 Participatory learning: Socratic reasoning chain

Teacher role. The teacher circulates, observes selected interactions, and intervenes if the AI gives premature, inaccurate, or unsafe advice.

Learner use of AI. The learner works through the case one question at a time and revises the clinical reasoning chain.

Illustrative exchange.

- **AI:** Which findings indicate cerebral dysfunction, and which indicate meningeal irritation?
- **Learner:** Confusion, drowsiness, and the focal seizure indicate cerebral dysfunction; severe headache and neck stiffness indicate meningeal irritation.
- **AI:** What reversible bedside cause of altered consciousness should still be checked immediately?
- **Learner:** Hypoglycaemia; the capillary glucose is normal in this case.
- **AI:** What disposition is required, and what must not be delayed while the diagnostic pathway is organized?
- **Learner:** Immediate emergency management with airway and physiological assessment, urgent senior review, and activation of the local central nervous system infection pathway.
- **AI:** Which investigations or treatment decisions must remain under teacher and guideline authority?
- **Learner:** The sequence of neuroimaging, lumbar puncture when safe, and empiric treatment should follow the local protocol and senior clinical judgment.

Teacher checkpoint. Specific antimicrobial regimens, procedural timing, contraindications, and local pathway details remain under teacher and guideline authority.

11.5 Post-assessment: non-graded individualized feedback

Teacher role. The teacher gives a new but conceptually related case and retains responsibility for any formal marking.

Learner use of AI. The learner submits a complete response and requests structured, non-graded feedback using the standardized reasoning spine.

Illustrative output. The AI identifies one strength, such as recognizing that persistent altered consciousness is unsafe for routine observation; one omission, such as failing to connect fever and neck stiffness with the seizure; and one priority for improvement, such as stating stabilization and emergency escalation before listing investigations.

Teacher checkpoint. The teacher reviews recurrent errors across learners and provides the authoritative whole-class explanation.

11.6 Summary and reflection

Teacher role. The teacher asks the learner to produce the summary first and checks the final product for clinical accuracy.

Learner use of AI. The learner drafts a localization map, red-flag checklist, triage pathway, initial-management checklist, and a short reflection before asking the AI to refine them.

Illustrative output. Example reflection: “I initially attributed the drowsiness only to the seizure. In a future similar case, I will look for preceding fever, headache, confusion, and meningeal signs, check immediate reversible causes, escalate urgently, and follow the local central nervous system infection pathway.”

Teacher checkpoint. The teacher corrects any oversimplified or locally inappropriate statement before the summary is retained in the learner log.
