# Supplementary material for: Faculty-supervised AI-supported BOPPPS-CBL and performance on a simulated neurological-emergency assessment among junior-college medical interns: a batch-stratified randomized educational study
Source: Front Med (Lausanne). 2026 Oct 14;13:1945397. doi: 10.3389/fmed.2026.1945397 (PMC13713343; doi:10.3389/fmed.2026.1945397)
Supplement: Supplementary file 2 [file Datasheet2.pdf]

Four-station neurological emergency OSCE: blueprint, scoring rubric, parallel case bank, and standardized-patient scripts

English reporting version. The assessment was administered in Chinese; this appendix provides the English rendering used for reporting and peer review.

Purpose. This appendix operationalizes the principal outcome described in Methods Sections 2.10 and 2.12 and contains the complete assessment framework: the OSCE blueprint, scoring rules, parallel Forms A and B, reserve vignettes, and Station 4 standardized-patient (SP) scripts. Each station contributes 25 points (18 analytic points plus a shared 0-7 global rating). The newly developed study OSCE is a new neurological-emergency recognition and triage instrument; the team's previous four-station neurology OSCE provides historical background only. The revised blueprint, cases, anchors, Chinese materials, result cards, and video clips were reviewed by five neurologists and pilot-tested in 15 learners. The study-use version was finalized in May 2025, and no modification to the scoring rubric was required after pilot testing.

1. Blueprint and administration

Station	Construct	Fixed stimulus	Coverage	Points
1	Red-flag recognition	Three short vignettes; structured written response	Stroke; severe headache/haemorrhage; seizure/status; dizziness	25
2	Lesion localization	Two vignettes; structured written response	Cortex/subcortex; brainstem; spinal cord; peripheral nerve; muscle/NMJ; central vs peripheral dizziness	25
3	Emergency triage and referral	Two vignettes; structured written response	Disposition, immediate action, and time target across modules	25
4	Neurological examination integration	One SP encounter plus standardized result card/video	Focused examination, localization, emergency interpretation, and disposition	25
Total				100

Parallel forms, shared anchor cases, and security. Forms A and B were alternated across consecutive monthly rotation batches. Form A was administered in batches 1, 3, 5, 7, 9, and 11, and Form B was administered in batches 2, 4, 6, 8, 10, and 12. Both randomized groups within each batch completed the same form. Each form contained five scored vignette cases, one standardized-patient script, and one reserve vignette. Assessment cases, wording, images, videos, and standardized-patient scripts were not used in teaching, formative quizzes, AI prompt demonstrations, or practice sessions. Because form assignment was determined by rotation batch, comparisons between forms were descriptive and did not constitute a formal equivalence assessment.

Administration. Candidates complete the four stations in a fixed sequence. Each station lasts exactly 10 minutes, with 2 minutes for transfer and instructions. Stations 1-3 use standardized written stimuli and response sheets; Station 4 is a live SP encounter. Candidates are identified only by study code, and no allocation information appears on assessment materials. Instructions, time limits, result cards, and scoring sheets are identical across randomized arms within each batch.

Station 4 standardization. Candidates demonstrate the relevant examination manoeuvre on the SP. Findings that a healthy SP cannot reliably simulate - including pathological plantar responses, direction-changing nystagmus, skew deviation, and head-impulse results - are supplied only after the candidate performs or explicitly requests the appropriate manoeuvre, using a standardized card or short video. The candidate must interpret and integrate the supplied finding into localization and triage.

2. Scoring, safety rules, and quality assurance

Scoring and adjudication. Two assessors blinded to allocation independently score every station. The station score is the mean of the two assessor totals, and the principal OSCE score is the sum of the four station means. A difference greater than 4 points on any station triggers independent scoring by a third blinded assessor; the final station score is the mean of the two closest totals. All assessors complete a calibration workshop using common written or video samples. No station assessment showed a between-assessor difference greater than 4 points; consequently, third-assessor scoring was not required for any of the 472 station assessments.

Partial credit and unsafe errors. Each analytic element has a predefined point allocation. Full points require all stated criteria; partial points are awarded for incomplete but correct content. An unsafe error is a recommendation that would discharge, substantially delay, or omit immediate stabilization of a clearly time-critical emergency. It yields 0 for the affected analytic element and caps that station's global rating at 3, without erasing unrelated correct elements.

Measurement quality. Inter-rater reliability was assessed using a two-way random-effects, absolute-agreement ICC for the mean of two assessors (ICC[2,2]). The ICC(2,2) was 0.82 for the total OSCE score and 0.85 for station-level ratings pooled across all four stations. Separate station-specific ICCs were not estimated. No station assessment required third-assessor scoring. Internal consistency across stations was summarized descriptively using Cronbach’s alpha because the stations assessed distinct but complementary constructs. Expert review focused on relevance, clarity, clinical accuracy, and construct alignment. Pilot testing examined feasibility, score distributions, floor and ceiling effects, apparent differences between assessment forms, and ambiguous scoring criteria. Detailed measurement results and score characteristics are presented in Supplementary Table 3.

2.1 Shared global rating scale (0-7)

Score	Anchor descriptor
0	No coherent approach; fails to recognize the emergency and proposes unsafe management.
1	Minimal relevant information; major unsafe omissions; no defensible disposition.
2	Recognizes some cues but misses a key danger feature or recommends a major delay.
3	Partially organized reasoning; emergency concern is present, but important gaps threaten timely care.
4	Safe minimum performance; recognizes the emergency and selects an appropriate disposition, with notable reasoning gaps.
5	Generally sound and prioritized; minor omissions do not alter immediate safety or disposition.
6	Fluent, well-prioritized integration of cues, localization, urgency, and next action; only trivial omissions.
7	Exceptionally coherent, efficient, and safe reasoning; explicitly justifies thresholds and excludes relevant alternatives.

2.2 Station-specific analytic rubrics

Station 1. Red-flag recognition (25 points)

Stimulus/construct: Three vignettes; candidate identifies danger cues, emergency categories, discrimination from plausible mimics, and urgency.

Element	Full-credit and partial-credit anchor	Score range
1A	Red-flag cue: 2 points per vignette (2 complete; 1 partly correct; 0 absent/incorrect).	0-6
1B	Emergency category: 2 points per vignette (2 appropriate time-critical category; 1 broad but safe; 0 incorrect/unsafe).	0-6
1C	Discrimination: 1 point per vignette for a feature distinguishing the emergency from a plausible mimic.	0-3
1D	Urgency rationale: 1 point per vignette for a relevant time window, deterioration risk, or non-delay reason.	0-3
GRS	Shared global rating above.	0-7

Station 2. Lesion localization (25 points)

Stimulus/construct: Two vignettes; candidate localizes along the neuraxis, links findings to level, and excludes a plausible alternative.

Element	Full-credit and partial-credit anchor	Score range
2A	Correct lesion level: 3 points per vignette (3 correct and sufficiently specific; 2 correct broad level; 1 partly correct; 0 incorrect).	0-6
2B	Justification: 3 points per vignette for linking at least two specific signs to the localization.	0-6
2C	Lateralization/tract: correct where applicable (partial credit for incomplete specification).	0-3
2D	Alternative exclusion: correct distinguishing feature with explicit exclusion of the main alternative.	0-3
GRS	Shared global rating above.	0-7

Station 3. Emergency triage and referral (25 points)

Stimulus/construct: Two vignettes; candidate selects disposition and prioritizes immediate action and timing.

Element	Full-credit and partial-credit anchor	Score range
3A	Disposition: 3 points per vignette (3 correct level; 1-2 directionally safe but imprecise; 0 unsafe).	0-6
3B	Threshold justification: 2 points per vignette for explaining why the selected level is required.	0-4
3C	Immediate first action: 2 points per vignette; action must be prioritized and must not delay stabilization.	0-4
3D	Time target/non-delay statement: 2 points per vignette.	0-4
GRS	Shared global rating above.	0-7

Station 4. Neurological examination integration (25 points)

Stimulus/construct: One SP encounter; candidate examines, requests/interprets standardized findings, integrates localization and emergency category, and states disposition.

Element	Full-credit and partial-credit anchor	Score range
4A	Technique: six predefined manoeuvre groups; 1 point each when relevant, correctly sequenced, and safely performed.	0-6
4B	Key abnormalities: identifies essential observed or card/video-provided findings; partial credit by finding.	0-4
4C	Integration: correct localization and likely emergency, explicitly linked to the findings.	0-4
4D	Disposition: correct urgency, immediate action, and non-delay statement.	0-4
GRS	Shared global rating above.	0-7

3. Parallel-form case bank

Use and study standardization. Candidate materials include only the stimuli; all scoring keys below are examiner material. Cases are simulated. Each scored form uses five vignettes; the reserve case was available only for substitution before study use. The standardized candidate materials, scoring keys, and SP scripts were used for the study and were not used in teaching or formative practice.

3.1 Parallel Form A

Case 1. Wake-up acute ischaemic stroke

Candidate stimulus: A 68-year-old woke 50 minutes ago with right facial droop, right arm weakness, and slurred speech. The patient was last known well immediately before sleep the previous night. Atrial fibrillation is present and no anticoagulant is taken. Blood pressure is 168/95 mmHg and capillary glucose is normal.

Scoring key (examiner material only):

- **Cues/classification:** Sudden focal deficit, wake-up/unknown onset, last-known-well, and atrial fibrillation; acute ischaemic stroke is likely, but the vignette alone does not establish large-vessel occlusion.
- **Action: Activate the stroke pathway; obtain urgent non-contrast CT; assess vascular/advanced imaging and reperfusion eligibility according to the local pathway; document glucose and last-known-well.**
- **Safety/discrimination:** Do not delay imaging or stroke-team assessment and do not substitute time noticed for last-known-well. Exclude hypoglycaemia; isolated lower-motor-neuron facial weakness would not explain arm weakness and dysarthria.

Case 2. Thunderclap headache suspicious for subarachnoid haemorrhage

Candidate stimulus: A 45-year-old reports a sudden "worst headache of life" that reached maximum intensity within seconds 2 hours ago, with vomiting, neck stiffness, and photophobia. The patient is alert and has no focal deficit.

Scoring key (examiner material only):

- **Cues/classification:** Instantaneous maximal onset, meningism, and vomiting; suspected subarachnoid haemorrhage.
- **Action: Urgent non-contrast CT head and emergency specialist pathway. If CT is negative, further testing follows CT timing/quality and the local guideline; when CT is obtained more than 6 hours after onset and suspicion persists, lumbar puncture may be required and is timed at least 12 hours after onset.**
- **Safety/discrimination:** Do not delay imaging or discharge as benign headache solely because the neurological examination is normal. Gradual build-up or a recurrent identical benign pattern lowers suspicion but does not override a true thunderclap history.

Case 3. Acute vestibular syndrome: central versus peripheral

Candidate stimulus: 3A: A 60-year-old with vascular risk factors has continuous vertigo for 3 hours, new dysarthria, and inability to walk unaided. A trained examiner documents a normal head-impulse test, direction-changing gaze-evoked nystagmus, and vertical skew. 3B: A 38-year-old has continuous vertigo for 12 hours after a viral illness, nausea, and unsteadiness but can walk with assistance. A trained examiner documents an abnormal unilateral head-impulse test, unidirectional horizontal nystagmus, no skew, and no focal neurological sign.

Scoring key (examiner material only):

- **Classification:** 3A is a central acute vestibular syndrome concerning for posterior-circulation stroke; 3B is a peripheral acute vestibular syndrome most consistent with vestibular neuritis, not BPPV because symptoms are continuous rather than brief and position-triggered.
- **Action:** 3A requires an emergency stroke pathway and appropriate urgent imaging; 3B receives clinical treatment/follow-up according to local practice after central features are excluded.
- **Safety/discrimination:** HINTS is used only for an acute continuous vestibular syndrome with spontaneous nystagmus and by a trained examiner; brief positional attacks require positional testing.

Case 4. Convulsive status epilepticus

Candidate stimulus: A 24-year-old with epilepsy and a missed antiseizure dose is in a generalized convulsion that has continued for 7 minutes and did not regain awareness between two earlier episodes.

Scoring key (examiner material only):

- **Cues/classification:** Continuous convulsion for at least 5 minutes or recurrent seizures without recovery; convulsive status epilepticus.
- **Action:** Protect from injury, position and support airway/breathing, check glucose, and administer a first-line benzodiazepine by the fastest locally recommended route. Escalate according to the status-epilepticus protocol if seizures continue.
- **Safety/discrimination:** Obtaining intravenous access must not delay treatment when another recommended route is immediately available. A single self-terminating seizure followed by recovery is not status epilepticus.

Case 5. Acute spinal cord compression

Candidate stimulus: A 55-year-old with malignancy reports 1 day of progressive bilateral leg weakness, a sensory level at the umbilicus, urinary retention, and back pain.

Scoring key (examiner material only):

- **Localization/classification:** Spinal cord, supported by bilateral leg involvement, a sensory level, and sphincter dysfunction; acute spinal cord compression, including metastatic compression.
- **Action:** Emergency whole-spine MRI and immediate specialist referral according to the local cord-compression pathway.
- **Safety/discrimination:** Do not delay imaging or definitive referral because neurological function may become irreversible. Glove-and-stocking loss suggests peripheral neuropathy; a unilateral cortical pattern would not produce a truncal sensory level.

3.2 Parallel Form B

Case 7. Acute intracerebral haemorrhage pattern

Candidate stimulus: A 62-year-old develops sudden left-sided weakness and reduced alertness with vomiting and severe headache. Blood pressure is 224/118 mmHg and capillary glucose is normal.

Scoring key (examiner material only):

- **Cues/classification:** Sudden focal deficit, reduced consciousness, vomiting, severe headache, and markedly elevated blood pressure; acute stroke with intracranial haemorrhage strongly suspected, but imaging is required to distinguish haemorrhagic from ischaemic stroke.
- **Action:** Immediate stroke/emergency pathway, airway and physiological stabilization, and urgent non-contrast CT.
- **Safety/discrimination:** Do not delay stabilization or imaging and do not give antithrombotic therapy before haemorrhage is excluded. Blood pressure and headache raise suspicion but do not replace imaging.

Case 8. Thunderclap headache with delayed negative CT

Candidate stimulus: A 39-year-old had a headache that reached maximum intensity instantly 9 hours ago. Non-contrast CT is reported as negative, but neck stiffness and persistent severe pain remain.

Scoring key (examiner material only):

- **Cues/classification:** True thunderclap onset with persistent clinical suspicion; subarachnoid haemorrhage remains possible despite a negative CT obtained more than 6 hours after onset.
- **Action:** Continue the emergency diagnostic pathway; consider lumbar puncture according to local guidance, timed at least 12 hours after symptom onset.
- **Safety/discrimination:** Do not reassure or discharge solely because the delayed CT is negative. A CT obtained within 6 hours and formally reported by an appropriately experienced radiologist follows a different pathway under some guidelines.

Case 9. Brief position-triggered vertigo

Candidate stimulus: A 36-year-old has recurrent spinning lasting less than 30 seconds when rolling to the right in bed. There is no continuous dizziness, no focal neurological symptom, and gait is normal between episodes. Right Dix-Hallpike testing provokes transient torsional up-beating nystagmus with latency and fatigability.

Scoring key (examiner material only):

- **Classification/localization:** Triggered episodic vestibular syndrome, typical of posterior-canal BPPV; peripheral vestibular localization.
- **Action:** Non-emergency management if no central red flags are present; perform an appropriate canalith-repositioning manoeuvre such as Epley.
- **Safety/discrimination:** Do not use HINTS because symptoms are not a continuous acute vestibular syndrome. Persistent direction-changing nystagmus, skew, severe gait failure, or focal neurological signs require a central pathway.

Case 10. Myasthenic respiratory crisis

Candidate stimulus: A 48-year-old with myasthenia gravis develops rapidly worsening dysphagia, weak cough, nasal speech, and breathlessness. Limb sensation is normal and pupils are equal and reactive.

Scoring key (examiner material only):

- **Cues/localization:** Bulbar weakness, weak cough, and respiratory symptoms; neuromuscular-junction localization supported by preserved sensation and pupils.
- **Classification/action:** Impending myasthenic crisis; immediate respiratory assessment and monitored emergency management with urgent neurology/critical-care involvement.
- **Safety:** Do not delay airway/ventilatory assessment; apparent oxygen saturation alone may not reveal early ventilatory failure.

Case 11. Guillain-Barre syndrome with respiratory risk

Candidate stimulus: A 31-year-old develops progressive symmetric ascending weakness and areflexia 10 days after diarrhoea. Facial weakness and shortness of breath are now present; sensation is mildly altered distally.

Scoring key (examiner material only):

- **Localization/classification:** Peripheral nerves/roots, supported by ascending symmetric weakness and areflexia; Guillain-Barre syndrome with respiratory/bulbar risk.
- **Action:** Hospital admission, serial respiratory-function assessment, autonomic monitoring, and urgent neurology review.
- **Safety/discrimination:** Do not delay respiratory assessment and escalation if function deteriorates. A sensory level or upper-motor-neuron signs would suggest spinal cord disease.

3.3 Reserve replacement vignettes

Status. Reserve cases were not included in the scored study forms unless substituted before study use.

Form A reserve - Case 6. Brainstem syndrome with crossed sensory signs

Candidate stimulus: A 70-year-old develops sudden left facial numbness, right-sided limb sensory loss, vertigo, hoarseness, dysphagia, and gait ataxia.

Scoring key: Crossed facial/body sensory signs with bulbar/cerebellar features localize to the brainstem; suspect posterior-circulation stroke and activate the urgent stroke/imaging pathway. Do not over-specify a named syndrome unless the complete pattern supports it.

Form B reserve - Case 12. Pontine crossed motor syndrome

Candidate stimulus: A 67-year-old develops sudden right lower-motor-neuron facial weakness with left arm and leg weakness and dysarthria.

Scoring key: Ipsilateral cranial-nerve-pattern facial weakness plus contralateral limb weakness localizes to the brainstem, most likely pons; activate the urgent posterior-circulation stroke/imaging pathway. A cortical lesion more often causes contralateral lower facial and limb weakness on the same body side.

4. Standardized-patient scripts for Station 4

General instructions. SPs provide the opening line and disclose history only when specifically asked. They simulate only findings that can be reproduced safely and reliably. Non-simulatable neurological

findings are never feigned; they are supplied by a standardized examiner card or video after the candidate performs or requests the relevant manoeuvre.

Field	SP-A. Wake-up unilateral weakness (Form A)
Role/setting	A 66-year-old who woke about 1 hour ago with right-sided weakness and facial droop.
Opening line	"Doctor, my right side suddenly went weak this morning."
History when asked	Deficit present on waking; last completely well before sleep; no headache or trauma; atrial fibrillation and hypertension; no anticoagulant.
Affect/behaviour	Mildly anxious; occasional slurring; safely produces reduced right arm and leg effort according to the training script.
SP-simulated findings	Right lower-facial asymmetry, pronator drift, and reduced right arm/leg effort when tested.
Standardized result card/video	After the candidate tests plantar responses: "Right extensor plantar response; left flexor." The SP does not simulate this finding.
Do not volunteer	Exact last-known-well and atrial fibrillation unless specifically asked.

Field	SP-B. Acute continuous vertigo (Form B)
Role/setting	A 58-year-old with 4 hours of continuous spinning, vomiting, and inability to walk unaided.
Opening line	"The room will not stop spinning and I cannot walk."
History when asked	Sudden and continuous; not triggered by position; new severe unsteadiness; hypertension, diabetes, smoking, and intermittent diplopia.
Affect/behaviour	Nauseated and holding still; demonstrates truncal unsteadiness only under SP safety training.
SP-simulated findings	Severe gait/truncal instability; candidate demonstrates eye-movement, cover, and head-impulse techniques on the SP.
Standardized result card/video	After the relevant manoeuvres, show normal head-impulse result, direction-changing gaze-evoked nystagmus, and vertical skew.
Do not volunteer	Vascular risk factors and diplopia unless asked.

5. Form mapping and assessment security

Form	Station 1	Station 2	Station 3	Station 4
A	Cases 1, 2, and 4	Cases 3 and 5	Cases 1 and 4	SP-A: wake-up unilateral weakness
B	Cases 7, 8, and 10	Cases 9 and 11	Cases 7 and 10	SP-B: acute continuous vertigo

Examiner packets and deviations. Examiner packets contained the response sheets, point-level keys aligned with this appendix, SP training checklists, result cards/videos, and translated candidate instructions. Forms were secured until all batches completed assessment. Any suspected exposure or administration deviation was to be documented and reviewed by the study team.
