# Supplementary material for: Faculty-supervised AI-supported BOPPPS-CBL and performance on a simulated neurological-emergency assessment among junior-college medical interns: a batch-stratified randomized educational study
Source: Front Med (Lausanne). 2026 Oct 14;13:1945397. doi: 10.3389/fmed.2026.1945397 (PMC13713343; doi:10.3389/fmed.2026.1945397)
Supplement: Supplementary file 3 [file Datasheet3.pdf]

Post-rotation written examination
MCQ and key-feature questions | Participant form and examiner scoring guide

English reporting version. The assessment was administered in Chinese; this appendix provides the English rendering used for reporting and peer review.

Purpose. This instrument measures post-rotation neurological-emergency knowledge and case-based key-feature decision-making. The summed 0-100 score represents an additional end-of-rotation research outcome, separate from the four-station OSCE principal outcome. Basic neurological examination technique is assessed within OSCE Station 4 and is not duplicated here.

Administration. The examination is administered once after teaching is complete. Standardized study-use items and scoring keys are not used in teaching, formative quizzes, AI demonstrations, or practice materials. Only Part I is provided to students; Part II is retained by the research team and assessors.

Assessment blueprint

Component	Format	Score	Primary data field
MCQ knowledge test	25 single-best-answer items; five options each	0-50	MCQ_score50
Key-feature questions	5 cases; cue/localization (0-4) + action/triage (0-6) per case	0-50	KFQ_score50
End-of-rotation total	MCQ score + KFQ score	0-100	ExitExam_score100

Part I. Participant-facing assessment form

A. MCQ knowledge test (25 items; 50 points)

Mark one best answer for each item. Each correct answer is worth 2 points.

1. A patient presents with sudden focal weakness of known onset time. Which is the most appropriate first brain investigation?
□ A. MRI brain with contrast
□ B. Non-contrast CT head
□ C. Carotid ultrasound
□ D. EEG
□ E. Lumbar puncture

2. The main reason to establish the last-known-well time in acute stroke is to determine:
□ A. Need for antibiotics
□ B. Eligibility for time-dependent reperfusion pathways
□ C. Choice of analgesia
□ D. Need for EEG
□ E. Long-term rehabilitation placement

3. Which bedside test must be performed promptly in any patient with an acute focal neurological deficit?
□ A. Capillary blood glucose
□ B. Spirometry
□ C. Urine culture
□ D. Abdominal ultrasound
□ E. Audiometry

4. Sudden focal deficit in a patient with atrial fibrillation most strongly raises concern for:
□ A. Cardioembolic ischaemic stroke
□ B. Bell palsy
□ C. Tension headache
□ D. Peripheral neuropathy
□ E. Myopathy

5. A wake-up stroke was first noticed 30 minutes ago, but the patient was normal before sleep 8 hours ago. The correct time statement is:
□ A. Onset was definitely 30 minutes ago
□ B. Last-known-well was 8 hours ago
□ C. The time window is irrelevant
□ D. The patient is automatically ineligible for any acute pathway
□ E. Use the time of hospital arrival as onset

6. A headache that reaches maximum intensity within seconds is best described as:
□ A. Tension-type headache
□ B. Thunderclap headache
□ C. Medication-overuse headache
□ D. Sinus headache
□ E. Postural headache

7. A patient with thunderclap headache has a negative CT performed 9 hours after onset and clinical suspicion remains high. What is the appropriate diagnostic pathway?
□ A. Discharge without follow-up
□ B. Consider lumbar puncture according to the local pathway, timed at least 12 hours after onset
□ C. Repeat CT in one month
□ D. Start migraine prophylaxis
□ E. Order EEG

8. Sudden focal deficit with vomiting, reduced consciousness, severe headache, and markedly elevated blood pressure most strongly suggests:
□ A. Intracranial haemorrhage pending urgent CT confirmation
□ B. Benign positional vertigo
□ C. Peripheral neuropathy
□ D. Myasthenia gravis
□ E. Tension headache

9. Which finding is most concerning in a patient with severe headache?
□ A. Gradual mild pain after a long workday
□ B. Instantaneous maximal onset with neck stiffness
□ C. Pain relieved by rest
□ D. Long history of identical headaches with normal examination
□ E. Mild scalp tenderness

10. In an acute continuous vestibular syndrome with spontaneous nystagmus, which trained-examiner HINTS pattern is most concerning for a central cause?
□ A. Abnormal head impulse, unidirectional nystagmus, no skew
□ B. Normal head impulse, direction-changing nystagmus, skew present
□ C. Positive Dix-Hallpike with transient torsional nystagmus
□ D. Symptoms only when rolling in bed
□ E. Isolated tinnitus

11. Brief vertigo triggered by rolling in bed with a typical positive Dix-Hallpike test is most consistent with:
□ A. Posterior-canal benign paroxysmal positional vertigo
□ B. Vestibular neuritis
□ C. Status epilepticus
□ D. Spinal cord compression
□ E. Subarachnoid haemorrhage

12. Which cluster is a warning pattern for posterior-circulation ischaemia?
□ A. Dizziness, diplopia, dysarthria, dysphagia, and ataxia
□ B. Fever, rash, and arthralgia
□ C. Chest pain and dyspnoea
□ D. Polyuria and polydipsia
□ E. Isolated low-back pain

13. Convulsive status epilepticus is operationally defined as:
□ A. Any seizure lasting more than 30 seconds
□ B. Two seizures in one year
□ C. Continuous convulsion for 5 minutes or recurrent seizures without recovery
□ D. Any seizure with incontinence
□ E. A seizure followed by brief confusion

14. The first-line drug class for convulsive status epilepticus is:
□ A. Benzodiazepine
□ B. Beta-blocker
□ C. Mannitol
□ D. Aspirin
□ E. Calcium-channel blocker

15. During an ongoing generalized convulsion, which immediate action must not be delayed?
□ A. Check airway safety and blood glucose while preparing first-line benzodiazepine
□ B. Arrange outpatient EEG
□ C. Begin long-term rehabilitation planning
□ D. Perform lumbar puncture at the bedside
□ E. Discharge if the seizure stops within 20 minutes

16. Bilateral leg weakness with a sensory level and urinary retention localizes the lesion to the:
□ A. Cerebral cortex
□ B. Brainstem
□ C. Spinal cord
□ D. Peripheral nerve
□ E. Muscle

17. Ipsilateral facial signs with contralateral limb signs most strongly localize to the:
□ A. Cortex
□ B. Internal capsule
□ C. Brainstem
□ D. Peripheral nerve
□ E. Muscle

18. Hyperreflexia with an up-going plantar response indicates involvement of the:
□ A. Lower motor neuron
□ B. Upper motor neuron pathway
□ C. Sensory nerve only
□ D. Neuromuscular junction
□ E. Muscle

19. A symmetric glove-and-stocking sensory loss most strongly suggests:
□ A. Peripheral neuropathy
□ B. Spinal cord lesion
□ C. Brainstem stroke
□ D. Basal ganglia disease
□ E. Myopathy

20. Ascending symmetric weakness with areflexia after a recent infection most suggests:
□ A. Ischaemic stroke
□ B. Guillain-Barre syndrome
□ C. Early myasthenic crisis
□ D. Subarachnoid haemorrhage
□ E. Bell palsy

21. Progressive bilateral leg weakness, back pain, a thoracic sensory level, and urinary retention in a patient with malignancy require:
□ A. Urgent whole-spine MRI and urgent specialist referral
□ B. Outpatient vitamin testing only
□ C. Reassurance and follow-up in three months
□ D. Carotid ultrasound
□ E. Epley manoeuvre

22. A normal head-impulse test in an acute continuous vestibular syndrome should be interpreted as:
□ A. Reassuring for BPPV
□ B. A potential central red flag when combined with other danger signs
□ C. Evidence that no neurological examination is needed
□ D. Diagnostic of migraine
□ E. A reason to discharge without gait assessment

23. Pronator drift most directly indicates:
□ A. Upper-motor-neuron weakness
□ B. Pure sensory loss
□ C. Cerebellar tremor only
□ D. Normal variation in all patients
□ E. Peripheral vestibular dysfunction

24. A patient with acute focal deficit should not receive thrombolysis before:
□ A. Excluding intracranial haemorrhage on appropriate imaging
□ B. Carotid ultrasound is completed
□ C. EEG is completed
□ D. Two weeks of observation
□ E. Routine outpatient MRI

25. Which response best represents safe triage for sudden vertigo with inability to walk, diplopia, and direction-changing nystagmus?
□ A. Outpatient reassurance for peripheral vertigo
□ B. Emergency stroke pathway and urgent imaging
□ C. Epley manoeuvre only
□ D. Oral analgesia and home observation
□ E. Routine clinic review in two weeks

B. Key-feature questions (5 cases; 50 points)

For each case, identify the decisive cue or localization (4 points) and state the immediate next action or disposition (6 points). Each case is scored 0-10, and the five case scores are summed directly to a 0-50 KFQ score.

**KFQ 1. Acute focal weakness**
**Scenario:** A 72-year-old develops right-sided weakness and slurred speech. The symptoms were first noticed 40 minutes ago. The patient has atrial fibrillation and no anticoagulant use is documented.
**Question 1 (4 points): What single cue most determines immediate management urgency?**<br>Answer: ____________________
**Question 2 (6 points): What is the immediate next action or disposition?**<br>Answer: ____________________

**KFQ 2. Thunderclap headache**
**Scenario:** A 36-year-old reports a headache that reached maximum intensity within seconds 10 hours ago. Non-contrast CT is negative, but clinical suspicion remains high.
**Question 1 (4 points): Which cue means this patient should not simply be reassured and discharged?**<br>Answer: ____________________
**Question 2 (6 points): What is the immediate next action or disposition?**<br>Answer: ____________________

**KFQ 3. Acute continuous vertigo**
**Scenario:** A 64-year-old with hypertension has four hours of continuous vertigo, vomiting, diplopia, and inability to walk unaided. A trained examiner notes normal head impulse and direction-changing nystagmus.
**Question 1 (4 points): Is the pattern central or peripheral, and what is the decisive cue?**<br>Answer: ____________________
**Question 2 (6 points): What is the immediate next action or disposition?**<br>Answer: ____________________

**KFQ 4. Prolonged convulsion**
**Scenario:** A 19-year-old has been in a generalized convulsion for 8 minutes and has not regained consciousness between episodes.
**Question 1 (4 points): What label triggers immediate emergency action?**<br>Answer: ____________________
**Question 2 (6 points): What is the immediate next action or disposition?**<br>Answer: ____________________

**KFQ 5. Progressive bilateral leg weakness**

**Scenario:** A 55-year-old with a history of malignancy reports progressive bilateral leg weakness, back pain, a sensory level around the umbilicus, and urinary retention.
**Question 1 (4 points): Where is the lesion most likely located?**<br>Answer: ____________________
**Question 2 (6 points): What is the immediate next action or disposition?**<br>Answer: ____________________

Part II. Examiner and researcher scoring guide

1. MCQ answer key

Item	Ans	Item	Ans	Item	Ans	Item	Ans	Item	Ans
1	**B**	6	**B**	11	**A**	16	**C**	21	**A**
2	**B**	7	**B**	12	**A**	17	**C**	22	**B**
3	**A**	8	**A**	13	**C**	18	**B**	23	**A**
4	**A**	9	**B**	14	**A**	19	**A**	24	**A**
5	**B**	10	**B**	15	**A**	20	**B**	25	**B**

2. KFQ scoring key

Generic scoring rule. Cue/localization: 4 = correct and supported by the decisive feature; 2-3 = broadly correct but incomplete; 1 = minimally relevant; 0 = incorrect. Action/triage: 6 = correct, prioritized, and safe; 3-5 = directionally safe but incomplete; 1-2 = minimally appropriate but insufficiently prioritized; 0 = incorrect or unsafe. Clinically equivalent wording is accepted.

KFQ	Cue/localization key (0-4)	Action/triage key (0-6)	Unsafe response (0 for action)
**1**	Time since onset / last-known-well within a possible reperfusion pathway.	Check capillary glucose and activate the stroke pathway with urgent non-contrast CT and eligibility assessment.	No glucose/imaging; routine follow-up only.
**2**	Thunderclap onset / instantaneous maximal intensity, especially with persistent suspicion.	Follow the local SAH diagnostic pathway, including lumbar puncture timed at least 12 hours after onset if indicated.	Reassure and discharge despite thunderclap onset and persistent suspicion.
**3**	Central pattern: normal head impulse plus direction-changing nystagmus, diplopia, and gait failure.	Emergency stroke pathway with urgent imaging; MRI is preferred when posterior-circulation stroke is suspected and local resources permit.	Label as peripheral vertigo and discharge despite central signs/gait failure.
**4**	Convulsive status epilepticus: continuous convulsion for 5 minutes or recurrent seizures without recovery.	Protect airway and patient safety, check glucose, and give a first-line benzodiazepine by the fastest available recommended route.	Delay benzodiazepine until prolonged observation; unsafe positioning.
**5**	Spinal cord, suggested by bilateral leg involvement, sensory level, and sphincter dysfunction.	Urgent whole-spine MRI and urgent referral for possible spinal cord compression.	Outpatient review only despite suspected cord compression.

3. Scoring, data retention, and quality control

Variable	Range	Formula / meaning
MCQ_correct_n	0-25	Number of correct MCQ responses
MCQ_score50	0-50	MCQ_correct_n x 2
KFQ_score50	0-50	Sum of KFQ1-KFQ5 case scores (0-10 each)
ExitExam_score100	0-100	MCQ_score50 + KFQ_score50

- Retain item-level MCQ responses and KFQ cue/action scores; calculate component and total scores from raw fields rather than entering totals manually.
- Administer after all teaching is complete and before assessment feedback is released. Keep participant forms separate from examiner keys and scoring anchors.
- Protect item security across batches. Suspected leakage, broken assessor blinding, adjudication, or other deviations should be documented in the trial master file.
- Report ExitExam_score100 as the end-of-rotation written-examination total. Report MCQ_score50 and KFQ_score50 separately as explanatory component outcomes.
