# Supplementary material for: Faculty-supervised AI-supported BOPPPS-CBL and performance on a simulated neurological-emergency assessment among junior-college medical interns: a batch-stratified randomized educational study
Source: Front Med (Lausanne). 2026 Oct 14;13:1945397. doi: 10.3389/fmed.2026.1945397 (PMC13713343; doi:10.3389/fmed.2026.1945397)
Supplement: Supplementary file 4 [file Datasheet4.pdf]

Supplementary S4. Study questionnaires

English reporting version. The questionnaires were administered in Chinese; this appendix provides the English rendering used for reporting and review.

Two questionnaires were used in this study: (1) a brief *baseline questionnaire* administered on the first day of the neurology rotation, before allocation was disclosed to participants; and (2) a *post-rotation questionnaire* administered on the last day of the rotation.

I. Baseline questionnaire (all participants, pre-rotation)

Instructions: Please answer according to your actual situation before starting the neurology rotation. There are no right or wrong answers. Do not write your name. Your responses will be used only for teaching research and will not affect your rotation grade or evaluation. Mark one response for each statement.

Response scale

1	2	3	4	5
Strongly disagree	Disagree	Neutral	Agree	Strongly agree

1. Digital literacy

Item	Statement	1	2	3	4	5
**DIG01**	I am proficient in using smartphones, tablets, or computers to complete learning tasks, such as reading textbooks, watching instructional videos, or using medical apps.	☐	☐	☐	☐	☐
**DIG02**	I can efficiently search medical databases and search engines (e.g., UpToDate, Chinese Medical Journal Network, PubMed) for clinical-learning information.	☐	☐	☐	☐	☐
**DIG03**	I can judge whether medical content I find online is reliable, including recognizing whether the source is authoritative and whether the information is consistent with current guidelines.	☐	☐	☐	☐	☐
**DIG04**	I feel confident using platforms such as WeChat learning groups, Rain Classroom, or Tencent Meeting to communicate and collaborate with classmates and clinical teachers on learning tasks.	☐	☐	☐	☐	☐
**DIG05**	I am willing to learn and try new digital tools or technologies that could support my learning, such as AI-assisted learning tools or clinical decision-support software.	☐	☐	☐	☐	☐

Scoring: mean of the five items (range 1–5); higher scores indicate greater self-reported digital literacy.

Note: *The item wordings shown here reflect the domain coverage of the baseline digital-literacy questionnaire as administered (device proficiency, medical-information retrieval, content evaluation, digital communication, and openness to new digital tools). Individual item responses were aggregated into a mean score at data entry and were not preserved in the analytic dataset.*

II. Post-rotation questionnaire (all participants completed Part A on the last day of the rotation; the AI-supported group additionally completed Part B)

Instructions: Please answer according to your actual learning experience during this neurology rotation. There are no right or wrong answers. Do not write your name. Your responses will be used only for teaching research and will not affect your rotation grade or evaluation. Mark one response for each statement.

Response scale

1	2	3	4	5
Strongly disagree	Disagree	Neutral	Agree	Strongly agree

Part A. Questionnaire for all participants

Complete all 14 items in Sections A1–A3.

A1. Self-regulated learning

Item	Statement	1	2	3	4	5
**SRL01**	Before each learning session, I set specific learning goals.	☐	☐	☐	☐	☐
**SRL02**	When analysing clinical cases, I checked whether I truly understood the relevant content.	☐	☐	☐	☐	☐
**SRL03**	I was able to identify which clinical-reasoning steps I got wrong and analyse why I made those errors.	☐	☐	☐	☐	☐
**SRL04**	When performing lesion localization and emergency triage, I checked whether my reasoning was reasonable.	☐	☐	☐	☐	☐
**SRL05**	After receiving feedback from teachers, peers, or learning tools, I revised or adjusted my reasoning approach.	☐	☐	☐	☐	☐
**SRL06**	When I did not understand something, I actively sought explanation or clarification.	☐	☐	☐	☐	☐
**SRL07**	I adjusted my subsequent learning methods according to the errors I made during learning.	☐	☐	☐	☐	☐
**SRL08**	I evaluated my learning performance against the course learning objectives.	☐	☐	☐	☐	☐

A2. Learning satisfaction

Item	Statement	1	2	3	4	5
**SAT01**	The teaching structure and learning process of this course were clear and reasonable.	☐	☐	☐	☐	☐
**SAT02**	The teaching content of this course met my learning needs during the neurology clinical rotation.	☐	☐	☐	☐	☐
**SAT03**	Overall, I was satisfied with the teaching in this course.	☐	☐	☐	☐	☐

A3. Perceived learning burden

Item	Statement	1	2	3	4	5
**BUR01**	Completing the learning tasks in this course took up too much of my preparation time.	☐	☐	☐	☐	☐
**BUR02**	I felt considerable learning pressure while completing the learning tasks in this course.	☐	☐	☐	☐	☐
**BUR03**	The amount of learning tasks arranged in this course was too large for me.	☐	☐	☐	☐	☐

Part B. AI-assisted learning experience (AI-supported group only)

Complete this part only if you used the AI formative scaffold during the neurology rotation. Otherwise, please skip Part B.

B1. Perceived usefulness

Item	Statement	1	2	3	4	5
**AI_USE01**	The AI tool helped me analyse neurological-emergency cases in a more structured way.	☐	☐	☐	☐	☐
**AI_USE02**	The AI feedback helped me identify reasoning problems in lesion localization, red-flag recognition, or emergency triage.	☐	☐	☐	☐	☐
**AI_USE03**	Overall, the AI tool was helpful for my neurology learning.	☐	☐	☐	☐	☐

B2. Perceived ease of use

Item	Statement	1	2	3	4	5
**AI_EASE01**	I was able to learn how to use the AI tool quickly.	☐	☐	☐	☐	☐
**AI_EASE02**	The AI prompt templates and operating steps provided in this course were easy to understand.	☐	☐	☐	☐	☐
**AI_EASE03**	I was able to use the AI tool smoothly to complete the required learning tasks.	☐	☐	☐	☐	☐

B3. Trust in AI feedback

Item	Statement	1	2	3	4	5
**AI_TRUST01**	I considered the AI feedback on case reasoning to be generally accurate.	☐	☐	☐	☐	☐
**AI_TRUST02**	In most cases, the AI feedback was consistent with the teacher explanations and course requirements.	☐	☐	☐	☐	☐

B4. Concern about over-reliance

Item	Statement	1	2	3	4	5
**AI_OVER01**	When using AI, I sometimes sought hints before thinking sufficiently on my own.	☐	☐	☐	☐	☐
**AI_OVER02**	Without prompts from the AI tool, I might have difficulty independently reasoning through similar cases.	☐	☐	☐	☐	☐

B5. Future use intention

Item	Statement	1	2	3	4	5
**AI_INTENT01**	I would be willing to continue using similar AI tools in future medical learning.	☐	☐	☐	☐	☐
**AI_INTENT02**	I would recommend this form of AI-assisted learning to other interns.	☐	☐	☐	☐	☐

Thank you for completing the questionnaire.
