# Supplementary material for: Faculty-supervised AI-supported BOPPPS-CBL and performance on a simulated neurological-emergency assessment among junior-college medical interns: a batch-stratified randomized educational study
Source: Front Med (Lausanne). 2026 Oct 14;13:1945397. doi: 10.3389/fmed.2026.1945397 (PMC13713343; doi:10.3389/fmed.2026.1945397)
Supplement: Supplementary file 5 [file Table1.docx]

**Supplementary Table 1. Outcome hierarchy, multiplicity approach, and analysis status**

| **Outcome or analysis family** | **No. of tests** | **Analysis status** | **Multiplicity approach** |
| --- | --- | --- | --- |
| Four-station OSCE total score | 1 | Primary endpoint | Two-sided *α* = 0.05; no adjustment required for a single primary endpoint |
| Station-specific OSCE scores | 4 | Secondary domain-level analyses | Bonferroni-adjusted threshold, *P* < 0.0125 |
| Written examination total, MCQ component, and KFQ component | 3 | Secondary educational outcomes | No family-wise adjustment; estimates interpreted using 95% CIs without confirmatory claims across components |
| Self-regulated-learning items | 8 | Exploratory learner-reported outcome family | Bonferroni-adjusted threshold, *P* < 0.00625 |
| Satisfaction items | 3 | Exploratory learner-reported outcomes | No multiplicity adjustment; interpreted descriptively/exploratorily |
| Perceived-burden items | 3 | Exploratory learner-reported outcomes | No multiplicity adjustment; interpreted descriptively/exploratorily |
| Intervention-only AI-experience items | 12 | Descriptive, intervention group only | No between-group hypothesis testing |
| Assessment-form A/B score comparison | 1 | Exploratory descriptive comparison | No multiplicity adjustment; not interpreted as an equivalence test |
| Treatment × assessment-form interaction | 1 | Exploratory effect-modification analysis | No multiplicity adjustment |
| Alternative OSCE composites and covariate sensitivity analyses | 5* | Post hoc sensitivity analyses | No multiplicity adjustment; outside the primary inferential framework |
| Batch-stratified permutation analysis | 1 | Post hoc/randomization-based sensitivity analysis | No multiplicity adjustment |
| Treatment × digital-literacy interaction | 1 | Post hoc exploratory effect-modification analysis | No multiplicity adjustment |
| Treatment × baseline academic-performance interaction | 1 | Post hoc exploratory effect-modification analysis | No multiplicity adjustment |

**Note**: The alternative OSCE/covariate analyses comprised: additional adjustment for age and gender; exclusion of Station 3; the Stations 2+4 composite; Station 2 alone; and the Stations 1+3+4 composite excluding the lesion-localization station. Because no prospectively time-stamped statistical analysis plan was available, the chronology of analyses described as planned before outcome examination cannot be independently verified. Analyses developed after examination of the study data are explicitly labeled post hoc.

**Supplementary Table 2. Planned six-session instructional dose and available attendance records in both study arms**

| **Session** | **Planned neurological-emergency module** | **Scheduled contact time, AI-supported group** | **Scheduled contact time, conventional group** | **Attendance recorded, AI-supported / conventional** |
| --- | --- | --- | --- | --- |
| 1 | Acute ischemic stroke recognition and triage | 50–60 min | 50–60 min | 58/58; 60/60 |
| 2 | Hemorrhagic stroke and acute severe headache | 50–60 min | 50–60 min | 58/58; 60/60 |
| 3 | Acute dizziness and differentiation of central from peripheral vertigo | 50–60 min | 50–60 min | 58/58; 60/60 |
| 4 | Seizure, status epilepticus, and altered consciousness | 50–60 min | 50–60 min | 58/58; 60/60 |
| 5 | Acute limb weakness, numbness, and lesion localization across the neuraxis and neuromuscular system | 50–60 min | 50–60 min | 58/58; 60/60 |
| 6 | Integration of the neurological examination with referral decision-making | 50–60 min | 50–60 min | 58/58; 60/60 |
| **Total** | **Six scheduled sessions** | **300–360 min planned** | **300–360 min planned** | **348/348; 360/360 participant-sessions** |

**Note:** Two sessions were scheduled each week during the first three weeks of the rotation. Both groups shared the six module topics, learning objectives, scheduled contact time, and BOPPPS-CBL structure; the AI-supported group used standardized AI prompts under faculty supervision, whereas the conventional group received faculty and peer feedback. This table describes the intended scheduled dose and recorded attendance, not measured time spent on individual activities. Minutes spent in AI dialogue, reflection, learner preparation outside scheduled sessions, and activity-specific faculty contact were not recorded; the delivered activity-specific dose therefore cannot be quantified. Supplementary Table 3 is a reporting example created after data collection, rather than a record of one of these six scheduled modules.

**Supplementary Table 3. Illustrative worked example of AI-supported and conventional BOPPPS-CBL delivery in one neurological-emergency module**

**Illustrative module and case.** Recognition and triage of impending myasthenic crisis. A 24-year-old woman with known myasthenia gravis developed progressive dysphagia, nasal speech, bilateral ptosis, neck-flexor weakness, a weak cough, and dyspnoea after an upper-respiratory infection. She was alert, had no sensory deficit, and could count only to 10 in one breath. The learning task was to localize the deficit, identify bulbar and respiratory red flags, and determine the required urgency and disposition.

| **BOPPPS phase** | **Case-specific learning task and expected output** | **AI-supported BOPPPS-CBL** | **Conventional BOPPPS-CBL** |
| --- | --- | --- | --- |
| **Bridge-in** | Identify the first three priority cues and explain why the presentation may be time-critical. | The learner previews the trigger case. The AI asks one question at a time about tempo, bulbar symptoms, cough strength, single-breath count, and respiratory distress. It does not state the diagnosis or disposition. | The teacher presents the same trigger case and asks whole-group questions about progression, bulbar symptoms, and immediate danger signs. |
| **Objectives** | Translate the session goals into four performance tasks: localization, red-flag recognition, differential reasoning, and triage. | The AI reformulates teacher-approved objectives as a task list. The learner selects a personal focus, such as linking sensory sparing and fatigability to neuromuscular-junction localization. | The teacher presents the same objectives and asks learners to identify a personal learning focus before the case discussion. |
| **Pre-assessment** | Commit an initial localization and disposition before receiving feedback. | After the learner records an independent answer, the AI asks which findings support neuromuscular-junction localization and which findings suggest impending respiratory failure. It does not judge correctness or display an answer key. | The learner completes the same short pre-assessment. The teacher reviews common answers after all learners have committed their responses. |
| **Participatory learning** | Construct a prioritized reasoning chain and perform a focused examination: localization -> bulbar/respiratory red flags -> urgency -> first actions. | Using one-question-at-a-time Socratic prompts, the AI guides the learner to interpret fatigability, sensory sparing, weak cough, reduced single-breath count, and worsening dyspnoea; consider plausible alternatives; and justify urgent senior and critical-care assessment according to the local pathway. The teacher monitors the interaction and corrects inaccurate or unsafe output. | The teacher facilitates the same case discussion, demonstrates the focused examination, and provides teacher- and peer-based feedback on localization, red flags, and disposition. |
| **Post-assessment** | Apply the reasoning structure to a conceptually related but different vignette and state the single most urgent next step. | The learner submits a complete response. The AI provides non-graded feedback structured as one strength, one omission, and one priority for improvement, following the fixed reasoning spine. Formal marking remains with the teacher. | The learner completes the same vignette and receives structured formative feedback from the teacher using the same learning objectives. |
| **Summary** | Produce a localization map, respiratory/bulbar red-flag checklist, triage pathway, initial-action checklist, and brief reflection. | The learner drafts all outputs before asking the AI to refine their organization. The AI prompts reflection on the missed cue and the planned response to a future similar case. The teacher verifies clinical accuracy. | The learner drafts the same outputs. The teacher synthesizes key points, corrects misconceptions, and guides the final reflection. |

**Note:** This is an illustrative reporting example created after data collection to demonstrate how the standardized teaching sequence was operationalized; it was not used as intervention material or in the OSCE, MCQ, or KFQ assessments. Learners in the AI-supported arm were required to provide an independent initial response before receiving AI feedback. The AI did not grade performance or replace teacher judgment. The standardized Chinese prompt templates and English renderings are provided in Supplementary Appendix S1. AI = artificial intelligence; BOPPPS = Bridge-in, Objectives, Pre-assessment, Participatory Learning, Post-assessment, Summary; CBL = case-based learning.

**Supplementary Table 4. Development, pilot testing, reliability, and score characteristics of the four-station neurological emergency OSCE**

**A. Development, pilot testing, and reliability**

| **Domain** | **Measure** | **Result** |
| --- | --- | --- |
| **Expert review** | Panel | 5 experts, all with neurology backgrounds |
| **Expert review** | Review focus | Relevance, clarity, clinical accuracy, and alignment with the intended constructs |
| **Pilot testing** | Sample and timing | 15 learners; completed in May 2025 |
| **Pilot testing** | Scoring rubric after pilot testing | No modification was required |
| **Inter-rater reliability** | Total OSCE score, ICC(2,2) | 0.82 |
| **Inter-rater reliability** | Station-level ratings pooled across four stations, ICC(2,2) | 0.85 |
| **Scoring adjudication** | Station assessments requiring a third assessor | 0/472 |

**B. Score characteristics in the randomized sample**

| **Outcome** | **n** | Mean ± SD | **Median (IQR)** | **Observed range** | **Floor, n (%)** | **Ceiling, n (%)** |
| --- | --- | --- | --- | --- | --- | --- |
| **OSCE total score (0-100)** | 118 | 79.20 ± 7.93 | 79.00 (74.25-84.00) | 60-96 | 0 (0.0) | 0 (0.0) |
| **Station 1: red-flag recognition (0-25)** | 118 | 17.32 ± 4.16 | 17.00 (14.00-21.00) | 10-25 | 0 (0.0) | 4 (3.4) |
| **Station 2: lesion localization (0-25)** | 118 | 20.64 ± 3.80 | 22.00 (18.00-24.00) | 12-25 | 0 (0.0) | 13 (11.0) |
| **Station 3: emergency triage and referral (0-25)** | 118 | 20.25 ± 3.33 | 21.00 (17.00-23.00) | 14-25 | 0 (0.0) | 9 (7.6) |
| **Station 4: neurological examination integration (0-25)** | 118 | 20.99 ± 2.95 | 22.00 (19.00-23.00) | 14-25 | 0 (0.0) | 12 (10.2) |

**C. Parallel-form administration and exploratory comparison**

| **Measure** | **Form A** | **Form B** | **Comparison** |
| --- | --- | --- | --- |
| **Rotation batches** | 1, 3, 5, 7, 9, 11 | 2, 4, 6, 8, 10, 12 | Alternated by batch |
| **Participants, n** | 59 | 59 | — |
| **OSCE total score, mean ± SD** | 79.41 ± 9.03 | 79.00 ± 6.74 | Mean difference, 0.41 (95% CI, −2.50 to 3.31); p = 0.782 |
| **Treatment-by-form interaction** | — | — | −1.63 points (95% CI, −7.29 to 4.02); p = 0.568 |

**Note**: Form A was administered in odd-numbered batches and Form B in even-numbered batches. Both randomized groups within each batch completed the same form. Because assessment form was completely determined by rotation batch, the between-form comparison was descriptive and should not be interpreted as a formal equivalence test. The treatment-by-form interaction was estimated in the principal adjusted model containing rotation-batch fixed effects, baseline academic performance, and digital literacy. A negative interaction estimate indicates that the estimated intervention effect was smaller for Form B than for Form A.

**Supplementary Table 5. Post hoc sensitivity analyses addressing shared assessment cases and other exploratory analyses of the principal OSCE outcome**

| **Analysis** | **Outcome/model** | **Adjusted estimate (95% CI)** | **P value** | **Adjusted Cohen's d** |
| --- | --- | --- | --- | --- |
| **Primary four-station OSCE (reference)** | Stations 1+2+3+4; score 0-100 | 5.67 points (2.86 to 8.49) | <0.001 | 0.74 |
| **Excluding the shared-case triage station** | Stations 1+2+4; score 0-75 | 4.68 points (2.23 to 7.13) | <0.001 | 0.70 |
| **Nonshared-case station composite** | Stations 2+4; score 0-50 | 3.39 points (1.57 to 5.21) | <0.001 | 0.68 |
| **Localization station only** | Station 2; score 0-25 | 2.58 points (1.25 to 3.91) | <0.001 | 0.71 |
| **Excluding the localization station** | Stations 1+3+4; score 0-75 | 3.09 points (0.66 to 5.53) | 0.013 | 0.46 |
| **Additional covariate adjustment** | OSCE total; principal model plus age and gender | 5.46 points (2.63 to 8.29) | <0.001 | 0.71 |
| **Effect modification by digital literacy** | Treatment group x mean-centered digital-literacy score | 1.17 points per one-point increase (-1.92 to 4.27) | 0.454 | Not applicable |
| **Batch-stratified randomization analysis** | Primary four-station OSCE; 20,000 within-batch permutations | 5.67 (2.86–8.49) | < 0.001ᵃ | 0.74 |
| **Effect modification by baseline academic performance** | Treatment group × mean-centered baseline academic-performance score | 0.72 points per one-point increase (−0.22 to 1.66) | 0.132 | Not applicable |

**Note:** The primary four-station OSCE analysis is reproduced in this table solely as a reference and was not a post hoc analysis. All other analyses presented in this table were post hoc and were outside the primary inferential framework. The principal and alternative score models adjusted for rotation batch, baseline academic performance, and digital literacy. The additional covariate model also included age and gender. Adjusted Cohen’s d was calculated using the residual standard deviation from the corresponding linear-regression model. Station 3 reused two case contexts previously encountered in Station 1, whereas Stations 2 and 4 did not share assessment cases with each other. The analyses excluding Station 3 or restricting the outcome to Stations 2 and 4 produced estimates in the same direction and of a broadly similar standardized magnitude to the primary analysis. However, because these analyses were post hoc and were based on the same assessment dataset, they do not demonstrate that the estimated between-group difference was independent of Station 3 and do not eliminate possible recognition, cueing, or carry-over effects arising from the reused cases. The digital-literacy interaction model included treatment group, mean-centered digital literacy, their interaction, rotation batch, and baseline academic performance. The baseline-academic-performance interaction model included treatment group, mean-centered baseline academic performance, their interaction, rotation batch, and digital literacy. Positive estimates indicate higher simulated-assessment scores among participants assigned to the AI-supported BOPPPS-CBL package. CI = confidence interval; OSCE = objective structured clinical examination. ᵃThe adjusted mean difference, 95% CI, and adjusted Cohen’s d are taken from the principal adjusted linear-regression model. The P value was obtained from the batch-stratified randomization-based analysis using 20,000 within-batch permutations.

**Supplementary Table 6. Item-level post-rotation questionnaire outcomes**

| **Question** | **Group** | **Strongly disagree, n (%)** | **Disagree, n (%)** | **Neutral, n (%)** | **Agree, n (%)** | **Strongly agree, n (%)** | **Adjusted OR (95% CI)** | **P value** |
| --- | --- | --- | --- | --- | --- | --- | --- | --- |
| **SRL01. Set specific learning goals** | AI-BOPPPS | 0 (0.0) | 16 (27.6) | 19 (32.8) | 12 (20.7) | 11 (19.0) | 0.90 (0.47 to 1.74) | 0.759 |
|  | BOPPPS | 1 (1.7) | 12 (20.0) | 23 (38.3) | 11 (18.3) | 13 (21.7) |  |  |
| **SRL02. Checked whether the relevant content was truly understood** | AI-BOPPPS | 0 (0.0) | 18 (31.0) | 18 (31.0) | 11 (19.0) | 11 (19.0) | 0.80 (0.42 to 1.55) | 0.513 |
|  | BOPPPS | 0 (0.0) | 13 (21.7) | 24 (40.0) | 11 (18.3) | 12 (20.0) |  |  |
| **SRL03. Identified and analysed errors in clinical reasoning** | AI-BOPPPS | 0 (0.0) | 2 (3.4) | 20 (34.5) | 23 (39.7) | 13 (22.4) | 2.38 (1.21–4.71) | 0.012 |
|  | BOPPPS | 0 (0.0) | 11 (18.3) | 26 (43.3) | 11 (18.3) | 12 (20.0) |  |  |
| **SRL04. Checked whether localization and triage reasoning was reasonable** | AI-BOPPPS | 0 (0.0) | 18 (31.0) | 15 (25.9) | 13 (22.4) | 12 (20.7) | 1.04 (0.54 to 2.01) | 0.900 |
|  | BOPPPS | 0 (0.0) | 16 (26.7) | 21 (35.0) | 11 (18.3) | 12 (20.0) |  |  |
| **SRL05. Revised reasoning after receiving feedback** | AI-BOPPPS | 0 (0.0) | 2 (3.4) | 16 (27.6) | 20 (34.5) | 20 (34.5) | 3.21 (1.61 to 6.41) | <0.001 |
|  | BOPPPS | 0 (0.0) | 8 (13.3) | 28 (46.7) | 13 (21.7) | 11 (18.3) |  |  |
| **SRL06. Actively sought explanation or clarification** | AI-BOPPPS | 1 (1.7) | 13 (22.4) | 16 (27.6) | 13 (22.4) | 15 (25.9) | 1.10 (0.57 to 2.11) | 0.771 |
|  | BOPPPS | 1 (1.7) | 14 (23.3) | 16 (26.7) | 15 (25.0) | 14 (23.3) |  |  |
| **SRL07. Adjusted subsequent learning methods according to errors** | AI-BOPPPS | 0 (0.0) | 15 (25.9) | 15 (25.9) | 17 (29.3) | 11 (19.0) | 1.13 (0.59 to 2.16) | 0.721 |
|  | BOPPPS | 0 (0.0) | 16 (26.7) | 20 (33.3) | 12 (20.0) | 12 (20.0) |  |  |
| **SRL08. Evaluated performance against learning objectives** | AI-BOPPPS | 0 (0.0) | 18 (31.0) | 15 (25.9) | 13 (22.4) | 12 (20.7) | 1.40 (0.72 to 2.70) | 0.317 |
|  | BOPPPS | 0 (0.0) | 18 (30.0) | 22 (36.7) | 15 (25.0) | 5 (8.3) |  |  |
| **SAT01. Teaching structure and learning process were clear and reasonable** | AI-BOPPPS | 0 (0.0) | 6 (10.3) | 17 (29.3) | 18 (31.0) | 17 (29.3) | 0.95 (0.49 to 1.83) | 0.877 |
|  | BOPPPS | 0 (0.0) | 6 (10.0) | 18 (30.0) | 17 (28.3) | 19 (31.7) |  |  |
| **SAT02. Teaching content met learning needs during the rotation** | AI-BOPPPS | 0 (0.0) | 5 (8.6) | 17 (29.3) | 18 (31.0) | 18 (31.0) | 0.85 (0.44 to 1.66) | 0.642 |
|  | BOPPPS | 0 (0.0) | 5 (8.3) | 15 (25.0) | 21 (35.0) | 19 (31.7) |  |  |
| **SAT03. Overall satisfaction with the course** | AI-BOPPPS | 0 (0.0) | 5 (8.6) | 18 (31.0) | 17 (29.3) | 18 (31.0) | 1.12 (0.58 to 2.17) | 0.737 |
|  | BOPPPS | 0 (0.0) | 7 (11.7) | 18 (30.0) | 18 (30.0) | 17 (28.3) |  |  |
| **BUR01. Learning tasks took too much preparation time** | AI-BOPPPS | 2 (3.4) | 3 (5.2) | 24 (41.4) | 21 (36.2) | 8 (13.8) | 5.86 (2.81 to 12.22) | <0.001 |
|  | BOPPPS | 8 (13.3) | 21 (35.0) | 20 (33.3) | 8 (13.3) | 3 (5.0) |  |  |
| **BUR02. Experienced considerable learning pressure** | AI-BOPPPS | 17 (29.3) | 17 (29.3) | 18 (31.0) | 3 (5.2) | 3 (5.2) | 1.30 (0.67 to 2.51) | 0.433 |
|  | BOPPPS | 20 (33.3) | 19 (31.7) | 16 (26.7) | 3 (5.0) | 2 (3.3) |  |  |
| **BUR03. The amount of learning tasks was too large** | AI-BOPPPS | 18 (31.0) | 17 (29.3) | 18 (31.0) | 2 (3.4) | 3 (5.2) | 1.02 (0.53 to 1.97) | 0.945 |
|  | BOPPPS | 18 (30.0) | 19 (31.7) | 18 (30.0) | 3 (5.0) | 2 (3.3) |  |  |

**Note**: Percentages are calculated within each group. Adjusted odds ratios were estimated using proportional-odds logistic regression with rotation batch, baseline academic performance, and digital literacy included in each model; an OR > 1 indicates a greater likelihood of selecting a higher response category in the AI-BOPPPS group. Internal consistency was low for the study-developed self-regulated-learning (Cronbach's α = 0.37) and satisfaction (α = 0.59) subscales, and negative for the perceived-burden subscale (α = −0.17); items were therefore analysed separately, and no composite scores were interpreted.

**Supplementary Table 7. Diagnostic and sensitivity analyses for the 14 post-rotation questionnaire items**

| **Item** | **Original 5-category model B (SE)** | **P value** | **Parallel-lines P** | **Collapsed 3-category model B (SE)** | **P value** | **Parallel-lines P** | **Interpretation** |
| --- | --- | --- | --- | --- | --- | --- | --- |
| SRL01 | −0.103 (0.334) | 0.759 | 0.974 | −0.080 (0.346) | 0.817 | 0.168 | Similar null finding |
| SRL02 | −0.219 (0.335) | 0.513 | 0.506 | −0.221 (0.345) | 0.522 | 0.378 | Similar null finding |
| SRL03 | 0.868 (0.347) | 0.012 | 0.593 | 1.161 (0.380) | 0.002 | 0.539 | Estimate strengthened after category collapse; exploratory |
| SRL04 | 0.042 (0.335) | 0.900 | 0.993 | 0.120 (0.349) | 0.731 | 0.965 | Similar null finding |
| SRL05 | 1.166 (0.353) | <0.001 | 0.991 | 1.282 (0.386) | <0.001 | 0.805 | Robust to category collapse |
| SRL06 | 0.097 (0.332) | 0.771 | 0.995 | 0.021 (0.353) | 0.953 | 0.995 | Similar null finding |
| SRL07 | 0.119 (0.333) | 0.721 | 0.430 | 0.246 (0.349) | 0.481 | 0.230 | Similar null finding |
| SRL08 | 0.336 (0.335) | 0.317 | 0.255 | 0.199 (0.344) | 0.564 | 0.128 | Similar null finding |
| SAT01 | −0.052 (0.334) | 0.877 | 0.084 | −0.009 (0.373) | 0.981 | 0.861 | Similar null finding |
| SAT02 | −0.157 (0.337) | 0.642 | 0.133 | −0.242 (0.387) | 0.532 | **0.013** | Proportional-odds assumption not supported after collapse; binary sensitivity remained null |
| SAT03 | 0.113 (0.337) | 0.737 | 0.769 | 0.183 (0.386) | 0.635 | 0.210 | Similar null finding |
| BUR01 | 1.768 (0.375) | <0.001 | 0.384 | 1.904 (0.389) | <0.001 | 0.218 | Robust to category collapse |
| BUR02 | 0.263 (0.335) | 0.433 | 1.000 | 0.334 (0.379) | 0.379 | 0.890 | Similar null finding |
| BUR03 | 0.023 (0.335) | 0.945 | 1.000 | 0.056 (0.373) | 0.881 | 0.432 | Similar null finding |

**Note: B represents the treatment-group coefficient on the log-odds scale; positive values indicate greater odds of selecting a higher response category in the AI-supported BOPPPS-CBL group. All models included treatment group, rotation batch, baseline academic performance, and digital literacy. The original models used the five ordered Likert categories. For sensitivity analyses, responses were collapsed into three ordered categories: disagree (1–2), neutral (3), and agree (4–5). The proportional-odds assumption was assessed using the test of parallel lines. All original five-category models and 13 of 14 collapsed-category models showed no evidence against the proportional-odds assumption. For SAT02, the collapsed-category model showed evidence of non-proportional odds (parallel-lines P = 0.013); a binary logistic sensitivity analysis comparing agree/strongly agree versus all other responses yielded OR = 0.79 (95% CI, 0.36–1.73; P = 0.559). All fitted models converged without iteration or Hessian warnings. Complete original response-category counts and adjusted odds ratios are reported in Supplementary Table 6. SRL = self-regulated learning; SAT = satisfaction; BUR = perceived burden; SE = standard error.**

**Supplementary Table 8. Item-level response distributions for the AI-experience questionnaire (AI-supported group, n = 58)**

| **Item** | **Strongly disagree, n (%)** | **Disagree, n (%)** | **Neutral, n (%)** | **Agree, n (%)** | **Strongly agree, n (%)** |
| --- | --- | --- | --- | --- | --- |
| AI_USE01 | 3 (5.2) | 2 (3.4) | 18 (31.0) | 18 (31.0) | 17 (29.3) |
| AI_USE02 | 2 (3.4) | 3 (5.2) | 18 (31.0) | 18 (31.0) | 17 (29.3) |
| AI_USE03 | 3 (5.2) | 2 (3.4) | 17 (29.3) | 18 (31.0) | 18 (31.0) |
| AI_EASE01 | 2 (3.4) | 4 (6.9) | 17 (29.3) | 17 (29.3) | 18 (31.0) |
| AI_EASE02 | 3 (5.2) | 3 (5.2) | 17 (29.3) | 17 (29.3) | 18 (31.0) |
| AI_EASE03 | 3 (5.2) | 3 (5.2) | 18 (31.0) | 16 (27.6) | 18 (31.0) |
| AI_TRUST01 | 3 (5.2) | 3 (5.2) | 17 (29.3) | 17 (29.3) | 18 (31.0) |
| AI_TRUST02 | 3 (5.2) | 3 (5.2) | 18 (31.0) | 17 (29.3) | 17 (29.3) |
| AI_OVER01 | 4 (6.9) | 2 (3.4) | 18 (31.0) | 18 (31.0) | 16 (27.6) |
| AI_OVER02 | 3 (5.2) | 3 (5.2) | 17 (29.3) | 17 (29.3) | 18 (31.0) |
| AI_INTENT01 | 3 (5.2) | 3 (5.2) | 17 (29.3) | 17 (29.3) | 18 (31.0) |
| AI_INTENT02 | 3 (5.2) | 3 (5.2) | 17 (29.3) | 18 (31.0) | 17 (29.3) |

Note: Values are n (%) for the AI-supported group (n = 58); all participants answered each item. Item codes correspond to the 12 AI-experience questions in Supplementary Appendix S4, Part B, which provides the full item wording. Response options range from 1 (strongly disagree) to 5 (strongly agree). AI_OVER01 and AI_OVER02 assess concerns about reliance on AI prompting; higher agreement indicates greater concern. Percentages may not total 100.0% because of rounding.
